# Additions to the “Martian Flora”: new botanical records from the Mars Desert Research Station, Utah

**DOI:** 10.3897/BDJ.8.e55063

**Published:** 2020-08-18

**Authors:** Paul C. Sokoloff, David A. Murray, Samantha R.M. McBeth, Michael G. Irvine, Shannon M. Rupert

**Affiliations:** 1 Beaty Centre for Species Discovery, Canadian Museum of Nature, Gatineau, Canada Beaty Centre for Species Discovery, Canadian Museum of Nature Gatineau Canada; 2 Mars Society, Lakewood, United States of America Mars Society Lakewood United States of America; 3 Independent Researcher, Gatineau, Canada Independent Researcher Gatineau Canada; 4 Live It, Victoria, Canada Live It Victoria Canada

**Keywords:** Floristics, Utah, Mars analogue, botany

## Abstract

The Mars Desert Research Station (MDRS) is a Mars-simulation campus set in a Martian planetary analogue in southern Utah. Despite a long history of astrobiology research, collections-based taxonomic inventories of the macro-level biodiversity around the station are relatively new. This study serves to add to the initial vascular plant list published for the station in 2016, where 39 species were recorded for MDRS. Here we report 40 new species, two new taxa recorded only to genus and two species re-identified from our 2016 fieldwork, bringing the total number of taxa in the "Martian" flora to 79 species and two taxa recorded to genus.

## Introduction

The Mars Desert Research Station (MDRS) in south-eastern Utah (Fig. 1) is a Mars mission simulation centre owned and operated by the Mars Society in the deserts outside of Hanksville (Persaud et al. 2003). Teams composed of scientists, engineers, medical practitioners, journalists, artists, etc. rotate through this station during one-to-two-week missions, where they work to understand and mitigate the technical and psychological challenges that will come with crewed Martian exploration (Rai and Kaur 2012, Sawyer et al. 2012) and conduct field research to better understand this site as an analogue of our planetary neighbour (Foing et al. 2011).

The deserts surrounding the station, south of the San Rafael Swell, are a true geologic analogue to Mars (Stoker et al. 2011). As such, there is a strong history of astrobiological field campaigns based out of the station, examining the microbial diversity of this unique area and techniques we might use in the search for such life on Mars (Direito et al. 2011, Martins et al. 2011, Thiel et al. 2011). Until recent years, nearly all biological fieldwork at MDRS focused on microbial life; however, in 2014, Crew 143, a Mars Society supported mission, began work to take inventory of the vascular plant, lichen and algal biodiversity of MDRS (Sokoloff et al. 2016).

Though there is a long tradition of floristic work across Utah and there are comprehensive plant lists for many areas near MDRS, like the San Rafael Swell (Harris 1983) and Capitol Reef National Park (Fertig 2009) - see Sokoloff et al. (2016) for a complete synopsis - the work started by Crew 143 was the beginning of a checklist specific to MDRS. In addition to 13 lichen and 6 algae/cyanobacterial taxa, this study recorded 39 vascular plant species for MDRS (reported erroneously as 38 in that paper).

While this initial study provided a baseline to work from, these collections were made primarily in November, when many species might not be conspicuously flowering or fruiting. At that point in the year, annuals or taxa which only spend part of their life cycle above ground (geophytes, for example) might also be overlooked. With 491 taxa recorded for the San Rafael Swell (Harris 1983) and 887 recorded for Capitol Reef National Park (Fertig 2009), it is highly likely that many species that do occur in the MDRS area were not apparent to Crew 143 or not in a phenological state where a collection would have led to a definitive identification.

Crew 143 also operated as a Mars-mission simulation, where trips outside of the main station campus (the Hab) were tightly controlled as simulated extra-vehicular activities (EVAs). On these excursions, crews were limited to exploring pre-approved sites, with a small team for a short amount of time (to simulate the constraints of working on Mars), reducing the amount of botanical exploration time available to the team. Additionally, the crew was required to wear simulated spacesuits while outside the hab, reducing visibility and dexterity while collecting. While all these conditions serve as a realistic learning opportunity about how to undertake field science on another world, it likely reduced the number of taxa recorded during the rotation.

The objective of our current study is to continue to develop the vascular plant checklist for MDRS by filling in collecting gaps caused by the above limitations. By re-collecting previously explored sites in the spring (rather than late autumn), we aim to fill in phenological gaps in our existing dataset. By botanising new locations and microhabitats across the MDRS exploration area in a non-simulation mission, with a crew made up predominantly of biologists, we hope to add new vascular plant taxa to the "Martian" flora.

## Materials and Methods

**Figure 2.**
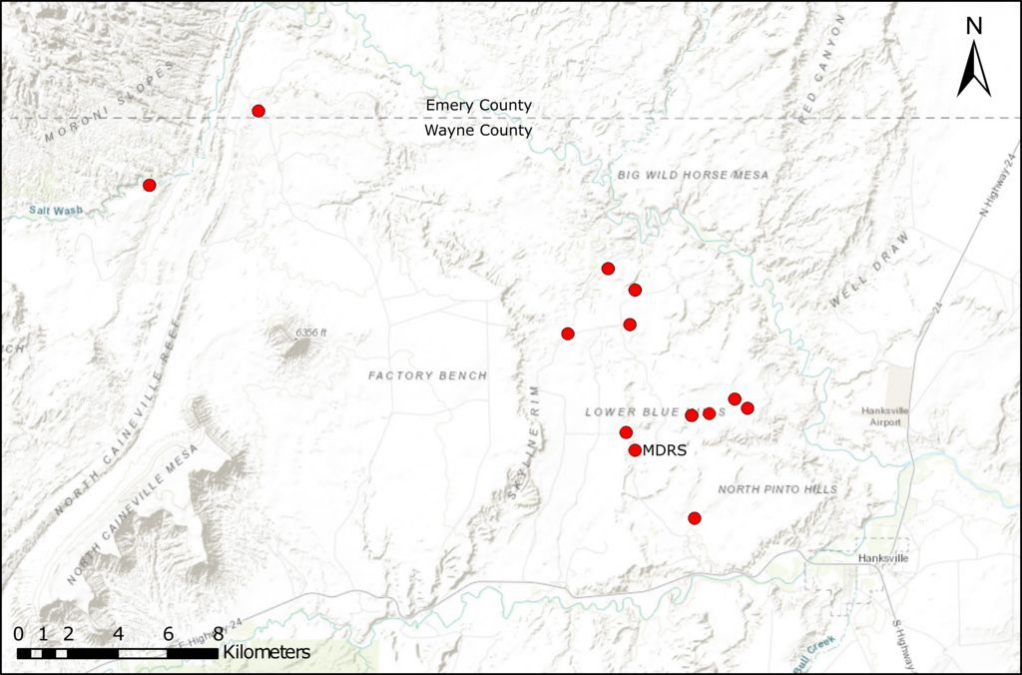
Collecting sites for the 2019 fieldwork carried out by Crew 210. Map Data: Esri, HERE, Garmin, Intermap, increment P Corp., GEBCO, USGS, FAO, NPS, NRCAN, GeoBase, IGN, Kadaster NL, Ordnance Survey, Esri Japan, METI, Esri China (Hong Kong), (c) Open Street Map contributors and the GIS User Community.

Crew 210, our biodiversity survey group, worked out of MDRS between 13-20 April 2019. During this week, our team made 63 vascular plant collections from 13 sites across the MDRS exploration area (Fig. 2, Table 1). We also made two new lichen collections (*Sokoloff et al. 1264* and *Sokoloff et al. 1323*) which are not treated in this manuscript.

At each sampling site, we surveyed the vegetation by searching various microhabitats on foot, seeking out plant taxa not previously documented from the station in Sokoloff et al. (2016). These plants were photographed *in situ* (where possible), dug up by the roots and placed in a plastic bag for transport back to MDRS, where they were pressed in the station's lab. Field notes - including coordinates, locality and habitat descriptions and a list of associated taxa - were recorded for each site for eventual transcription into specimen labels.

These herbarium specimens were identified using various literature sources, including the Flora of North America (Flora of North America Editorial Committee, eds. 1993), A Utah Flora (Welsh et al. 1993), the Atlas of North American Astragalus (Barneby 1964) and other publications as referenced in the annotated checklist below. A complete set of these voucher specimens have been deposited at the National Herbarium of Canada (CAN) at the Canadian Museum of Nature and duplicate specimens (as indicated in the specimen citations below) have been deposited at: the Intermountain Herbarium at the University of Utah (UTC), the Herbier Marie-Victorin at the Université de Montréal (MT) and the National Collection of Vascular Plants at Agriculture and Agri-Food Canada (DAO).

## Results

Of the 63 collections made by Crew 210, 12 were of taxa previously recorded from MDRS (Sokoloff et al. 2016), while the rest were vouchers for taxa newly recorded for the area (for our complete collection dataset, see Suppl. material 1). In addition to these specimens, we photo-documented one new species and two new genera for the study area, for a total of 42 newly-reported taxa for MDRS below the family level. Table 2 summarises these new records according to the linear sequence proposed by APG IV (The Angiosperm Phylogeny Group 2016); in the annotated checklist following, these taxa are listed alphabetically by family and species. Two Cactaceae specimens collected in 2016 have since been re-identified are also reported in the annotated checklist below.

The collecting sites visited during our rotation included a wider variety of habitats than our previous work in 2014, owing to the fact that Crew 210 was not working within the restraints of a Martian surface mission simulation and, therefore, had wider latitude to visit more sites each day and to go further afield. While complete habitat descriptions for all collecting sites can be found in Table 1, selected habitats are described below to illustrate a sample of the habitat diversity around the station.

In Emery county, north of MDRS, Factory Bench Road cuts through the "Valley of the Stars" (Fig. 3a), a gravelly scree slope with extensive gypsum deposits scattered across the eroded sandstone desert surface, where the minimal vegetative cover was dominated by *Artemisia tridentata*, *Ephedra viridis* and *Dasyochloa pulchella*. Further along the road, back in Wayne County, on the sandy shoreline of Salt Wash, a sheltered valley and water availability provide a sheltered microclimate where invasive *Tamarix ramosissima* and native *Ericameria nauseosa* thrive (Fig. 3b).

In Wayne County, east of the station, a sheltered, sandy wash provides adequate substrate and microclimate to host a unique local flora, including larger shrubs like Rhus trilobata subsp. trilobata and Populus fremontii subsp. fremontii (Fig. 3c). North of the station "Copernicus Valley" is a dry river valley with silty soils that host annual species like *Cleomella palmeriana* and Phacelia demissa var. demissa, alongside shrubby *Sarcobatus vermiculatus* (Fig. 3d). Immediately west of the station, "Hab Ridge" rises above MDRS, where a compacted capstone layer of the Dakota Sandstone formation is dominated by grasses (*Aristida purpurea*, for example) and shrubby species like *Gutierrezia sarothrae* (Fig. 3e, f).

## Annotated Checklist of New Vascular Plant Taxa

All vascular plant collections made by Crew 210 are included in the checklist below, which is arranged alphabetically by family and then species. Taxa previously collected at MDRS and recollected in 2019, are briefly listed under the family name. Taxa new to MDRS are listed under their own header, which includes the collection numbers corresponding to the species vouchers, any relevant notes about taxonomy, identification, and distribution, and if the taxon is present in one of four complete floristic inventories of locations close to MDRS: the San Rafael Swell (Harris 1983), Capitol Reef National Park (Fertig 2009), Glen Canyon National Recreation Area (Hill and Ayers 2009) and the Orange Cliffs region (Shultz et al. 1987).

### Amaranthaceae

We documented three new species in one new genus and one previously-documented genus for the MDRS area. We also made collections of species previously known from the station, including: *Atriplex confertifolia* (Torr. & Frém.) S. Wats. (*Sokoloff et al. 1317* [Fig. 4a], *1322*), Atriplex gardneri var. cuneata (A. Nelson) S.L. Welsh (*Sokoloff et al. 1281, 1302*) and *Kali tragus* (L.) Scop. (*Sokoloff et al. 1310*).

#### *Atriplex argentea* Nutt.

**Specimen Examined**: Utah, Wayne County: *Sokoloff et al. 1314* (CAN).

Found growing in silty sediment in Copernicus Valley, this species has been reported from Capitol Reef National Park (as var. argentea) (Fertig 2009), as uncommon in the San Rafael Swell (Harris 1983) and as uncommon in Glen Canyon National Recreation Area (Hill and Ayers 2009). Based on leaf shape, this specimen may be var. rydbergii following Welsh (2003), but as this collection is immature, we have only identified it to species.

#### Atriplex canescens (Pursh) Nutt. var. canescens

**Specimen Examined**: Utah, Wayne County: *Sokoloff et al. 1318* (CAN).

This shrub is widespread throughout Utah's deserts (Welsh 2003) and is common in the San Rafael Swell (Harris 1983), present in Capitol Reef National Park (Fertig 2009) and present in the Orange Cliffs region (Shultz et al. 1987). The nominate variety, var. canescens, is widespread throughout the species range, following Welsh (2003). In southern Utah and around MDRS, this species is distinguished by its four-winged fruiting bracteoles (Andersen 1996).

#### *Blitum nuttallianum* Schult.

**Specimen Examined**: Utah, Wayne County: *Sokoloff et al. 1320* (CAN, UTC).

This native annual species is commonly found on wetter, sub-alkaline clay (Holmgren 2003). This species was only recorded from the MDRS area in the immediate vicinity of the Hab, in lower areas where precipitation might accumulate in wet months (Fig. 4b). This species was previously recorded as occasional in the San Rafael Swell (Harris 1983), present in Capitol Reef National Park (Fertig 2009) and occasional in Glen Canyon National Recreation Area (Hill and Ayers 2009) as *Monolepis nuttaliana* (Schult.) Greene; however, recent phylogenetic work places this species in *Blitum* L. (Fuentes-Bazan et al. 2012).

### Amaryllidaceae

We documented one new species in one new genus for the MDRS area.

#### *Allium macropetalum* Rydb

**Specimens Examined**: Utah, Wayne County: *Sokoloff et al. 1271* (CAN); *1319* (CAN).

A common sight in the MDRS area (Fig. 4c), this species can be distinguished from the sympatric (and closely-related) *Allium textile* A.Nelson & J.F.Macbr. by its 3-5-veined spathe bracts (as opposed to 1-veined in *A. textile*) (McNeal and Jacobsen 2002). This species has been previously reported from Capitol Reef National Park (Fertig 2009), as uncommon in Glen Canyon National Recreational Area (Hill and Ayers 2009) and uncommon in the San Rafael Swell (Harris 1983).

### Anacardiaceae

We documented one new species in one new genus for the MDRS area.

#### Rhus trilobata Nutt. var. trilobata

**Specimen Examined**: Utah, Wayne County: *Sokoloff et al. 1297* (CAN, UTC).

This taxon was only encountered in the sheltered sandy wash south of BLM road 1104, with a unique local vegetation including Populus fremontii subsp. fremontii and *Symphoricarpos longiflorus* (Fig. 4d, e). Recorded as common in washes and roadsides in the San Rafael Swell (Harris 1983), the protected valley in the wash likely provides a warmer and less windy environment, providing an ideal microhabitat for multiple species not seen elsewhere near MDRS. This taxon has also been recorded from Capitol Reef National Park - as Rhus aromatica var. trilobata (Nutt.) A. Gray - (Fertig 2009) and as common in Glen Canyon National Recreation Area (Hill and Ayers 2009).

### Apiaceae

We documented one new species in one new genus for the MDRS area.

#### *Cymopterus glomeratus* (Nutt.) Raf.

**Specimen Examined**: Utah, Wayne County: *Sokoloff et al. 1273* (CAN, UTC).

This species was occasionally encountered throughout the MDRS area (Fig. 4f). This variable species has been reported from Capitol Reef National Park (Fertig 2009), as occasionally occurring in Glen Canyon National Recreation Area (Hill and Ayers 2009) (in both locations as Cymopterus acaulis var. fendleri (A. Gray) Goodrich), as present in the Orange Cliffs (Shultz et al. 1987) and common in the San Rafael Swell (Harris 1983) (in both locations as *Cymopterus fendleri* A. Gray). Current taxonomic concepts place both *C. acaulis* (including var. fendleri) and *C. fendleri* in *C. glomeratus* (Sun et al. 2005).

### Asparagaceae

We documented two new species in two new genera for the MDRS area.

#### *Eremocrinum albomarginatum* (M.E.Jones) M.E.Jones

**Specimen Examined**: Utah, Wayne County: *Sokoloff et al. 1289* (CAN, UTC).

This monotypic species was encountered sporadically on sandy soils east of MDRS (Fig. 5a). Endemic to the Colorado Plateau in southeast Utah and northern Arizona (Reveal and Utech 2002), this species has been reported from the San Rafael Swell, where it is common (Harris 1983), as present in Capitol Reef National Park (Fertig 2009) and from Glen Canyon National Recreation Area (Hill and Ayers 2009), where it is uncommon.

#### *Yucca harrimaniae* Trel.

**Specimen Examined**: Utah, Wayne County: *Sokoloff et al. 1298* (CAN).

This species was encountered infrequently, only on the top of "Hab Ridge" and in the sandy wash south of BLM road 1104 (Fig. 5b, c, d). Following Neese and Welsh (1985), these plants would be considered var. harrimaniae; however, recent treatments do not recognise varieties within this species (Hess and Robbins 2002). This species has been reported as common in the San Rafael Swell (Harris 1983), uncommon in Glen Canyon National Recreation Area (Hill and Ayers 2009) and present in Capitol Reef National Park (as var. harrimaniae) (Fertig 2009).

### Asteraceae

We documented four new species from four new genera for the MDRS area. We also made collections of species previously documented at the station, including: *Gaillardia spathulata* A. Gray (*Sokoloff et al. 1267*) and *Thelesperma subnudum* A. Gray (*Sokoloff et al. 1283*).

#### *Chaenactis stevioides* Hook. & Arn.

**Specimen Examined**: Utah, Wayne County: *Sokoloff et al. 1293* (CAN, UTC).

This species was not yet flowering when collected during our field season, but the characteristically hairy, dissected leaves made identification relatively straightforward (Morefield 2006). Found across the southwest United States (Andersen 1996), it is common in the San Rafael Swell (Harris 1983), present in Capitol Reef National Park (Fertig 2009), present in the Orange Cliffs area (Shultz et al. 1987) and widespread in Glen Canyon National Recreation Area (Hill and Ayers 2009). This species, along with its congener *Chaenactis fremontii* Gray, is believed to have arisen from independent aneuploid reduction events in *C. gabriuscula* DC. (Kyhos 1965).

#### *Malacothrix sonchoides* (Nutt.) Torr. & A. Gray

**Specimen Examined**: Utah, Wayne County: *Sokoloff et al. 1277* (CAN, UTC).

While our specimen was not flowering at the time of collection (Fig. 6a), we were able to identify this annual species on the basis of the species' distinctive leaf morphology (David 2006). It is common in the San Rafael Swell (Harris 1983), present in Capitol Reef National Park (Fertig 2009) and occasional in Glen Canyon National Recreation Area (Hill and Ayers 2009).

#### *Prenanthella exigua* (A. Gray) Rydb.

**Specimens Examined**: Utah, Emery County: *Sokoloff et al. 1263* (CAN). Wayne County: *Sokoloff et al. 1306* (CAN).

These young specimens possess toothed, black-spotted basal leaves which appear to be uncommon in the species (Fig. 6b); neither species accounts in the Flora of North America (Chambers 2006) nor A Utah Flora (Welsh et al. 1993) mention this trait and a search online (SEINet) only found two specimens with similar colouration (*Lehto 23527* [ASU 0103025] and *Williams 83-56-14* [NESH 81108]). It is reported as common in the San Rafael Swell (Harris 1983), present in Capitol Reef National Park (Fertig 2009), occasional in the Glen Canyon National Recreation Area (Hill and Ayers 2009) and present in the Orange Cliffs region (Shultz et al. 1987). Previously treated as *Lygodesmia exigua* A. Gray, Spencer Tomb (1972) re-established the species in *Prenanthella* (following Rydberg), based on chromosome number and pollen morphology. Molecular work by Lee et al. (2002) found this species to form a clade with *Pleiacanthus spinosus* (Nutt.) Rydb., another species formerly placed in *Lygodesmia*.

#### *Tetradymia glabrata* Torr. & A. Gray

**Specimen Examined**: Utah, Wayne County: *Sokoloff et al. 1316* (CAN, UTC).

Common in the San Rafael Swell (Harris 1983) and present in Capitol Reef National Park (Fertig 2009), this taxon is readily distinguished from other *Tetradymia* species in southern Utah by its spreading, club-shaped, glabrous secondary leaves (Fig. 6c, d) (Strother 1974, Strother 2006). This species is known to cause liver toxicity in livestock (Jennings et al. 1978).

### Boraginaceae

We documented six new species in three new genera and one previously-documented genus for the MDRS area.

#### Cryptantha crassisepala var. elachantha I.M. Johnst.

**Specimen Examined**: Utah, Wayne County: *Sokoloff et al. 1276* (CAN, UTC).

Variety *elachantha* is the more common infraspecific taxon of this annual species (Johnston 1959) and is the only variety present in Utah (Welsh et al. 1993). This taxon is common in the San Rafael Swell (Harris 1983), widespread in Glen Canyon National Recreation Area (Hill and Ayers 2009), present in Capitol Reef National Park (Fertig 2009) and present in the Orange Cliffs region as *Cryptantha crassisepala* s.l. (Shultz et al. 1987). This taxon is common throughout the MDRS operational area (Fig. 7a, b).

#### *Oreocarya flava* A.Nelson

**Specimens Examined**: Utah, Wayne County: *Sokoloff et al. 1275* (CAN, UTC); *1291* (CAN, UTC).

Common throughout southeast Utah (Higgins 1971, Payson 1927), this yellow-flowered species is distinctive in the MDRS area (Fig. 7c, d). Previously placed in *Cryptantha*, recent phylogenetic work has shown that genus to be polyphyletic and resurrected several genera previously submerged into *Cryptantha* as sections or subgenera (Hasenstab-Lehman and Simpson 2012, Mabry and Simpson 2018).

As *Cryptantha flava* (A. Nelson) Payson, this species has been reported as common in the San Rafael Swell (Harris 1983) and Glen Canyon National Recreation Area (Hill and Ayers 2009), as present in Capitol Reef National Park (Fertig 2009) and present in the Orange Cliffs region (Shultz et al. 1987).

#### *Oreocarya flavoculata* A. Nelson

**Specimen Examined**: Utah, Wayne County: *Sokoloff et al. 1268* (CAN, UTC).

Only encountered once in the MDRS area near Salt Wash (Fig. 7e, f), this species is common in the southwest United States (Higgins 1971). This species was also formerly placed in *Cryptantha*, until recent phylogenetic work confirmed the monophyly of *Oreocarya* (Hasenstab-Lehman and Simpson 2012, Mabry and Simpson 2018). This species is present in the Orange Cliffs Region (Shultz et al. 1987), occasional in Glen Canyon National Recreation Area (Hill and Ayers 2009), present in Capitol Reef National Park (Fertig 2009) and common in the San Rafael Swell (Harris 1983).

#### *Phacelia corrugata* A. Nelson

**Specimens Examined**: Utah, Emery County: *Sokoloff et al. 1260* (CAN, UTC). Wayne County: *Sokoloff et al. 1309* (CAN, UTC).

This species was encountered occasionally on rocky hilltops and raised clay swells in the MDRS area (Fig. 8a, b, c). Previously treated as Phacelia crenulata var. corrugata (A. Nelson) Brand (Welsh et al. 1993), molecular evidence supports the recognition of this taxon at the species level (Walden et al. 2014, Walden et al. 2016). This species is commmon in the San Rafael Swell (Harris 1983) and common in Glen Canyon National Recreation Area (Hill and Ayers 2009). *Phacelia crenulata* Torr. ex S. Watson is reported as present in the Orange Cliffs region (Shultz et al. 1987), though their taxonomic concept may have included *P. corrugata*.

#### Phacelia demissa A. Gray var. demissa

**Specimens Examined**: Utah, Wayne County: *Sokoloff et al. 1308* (CAN); *1312* (CAN, UTC).

Common on the silty flats of Copernicus Valley and on clay formations around the MDRS area (Fig. 8d, e), this species is endemic to the Four Corners States and Wyoming (Soreng et al. 1984). This taxon is present in Capitol Reef National Park (Fertig 2009), common in Glen Canyon National Recreation Area (Hill and Ayers 2009) and occasional in the San Rafael Swell (Harris 1983). Harris (1983) also reported Phacelia demissa var. heterotricha J.T. Howell from the San Rafael Swell; however, that variety is now considered a synonym of var. demissa (Welsh et al. 1993). Phacelia demissa var. minor N.D. Atwood is found outside the MDRS area in the Uintah Basin (Welsh et al. 1993).

#### *Tiquilia latior* (I.M. Johnston) A. Richards.

**Specimen Examined**: Utah, Wayne County: *Sokoloff et al. 1290* (CAN).

Only encountered once in the MDRS area on sandy plains (Fig. 8f), this species is endemic to the south-western United States and is found in Utah, Arizona and Nevada (Higgins 1979, Moore and Jansen 2007). This species is present in Capitol Reef National Park (Fertig 2009), common in Glen Canyon National Recreation Area (Hill and Ayers 2009) and occasional in the San Rafael Swell (Harris 1983).

### Brassicaceae

We documented four new species from four new genera for the MDRS area. We also made a collection of a species previously known from the station: *Lepidium montanum* Nutt. (*Sokoloff et al. 1304*).

#### *Chorispora tenella* (Pall.) DC.

This invasive weed was photographed (Fig. 9) in the vicinity of the Burpee Dinosaur Quarry north of MDRS, but was not collected at that time. It is common across the western United States (Rollins 1980) where it readily grows in disturbed areas, fields and along roadsides (Al-Shehbaz 2010d). This species has been reported from Capitol Reef National Park (Fertig 2009) and as uncommon in the San Rafael Swell (Harris 1983).

#### Descurainia pinnata subsp. brachycarpa (Richardson) Detling

**Specimens Examined**: Utah, Emery County: *Sokoloff et al. 1262* (CAN, UTC). Wayne County: *Sokoloff et al. 1303* (CAN).

Widespread throughout much of North America (Goodson and Al-Shehbaz 2010), this species was encountered sporadically on rocky ridges and hilltops (Fig. 9). *Descurainia pinnata* (not determined to subspecies) is reported as common in Glen Canyon National Recreation Area (Hill and Ayers 2009) and present in the Orange Cliffs Region (Shultz et al. 1987), while this taxon is reported as uncommon in the San Rafael Swell (Harris 1983) and present in Capitol Reef National Park (Fertig 2009) as Descurainia pinnata var. intermedia (Rydberg) C. L. Hitchcock - a synonym of subsp. brachycarpa (Goodson and Al-Shehbaz 2010).

#### *Stanleya pinnata* (Pursh) Britton var. pinnata

**Specimen Examined**: Utah, Wayne County: *Sokoloff et al. 1266* (CAN).

This well-known selenophyte (Feist and Parker 2001) was only encountered once in the MDRS area, near Salt Wash. Delimitation of varieties in this species has changed through time (Rollins 1939, Lichvar 1983, Turner 2004); here we follow Al-Shehbaz (2010a). This taxon is reported as widespread in Glen Canyon National Recreation Area (Hill and Ayers 2009). *Stanleya pinnata* is reported from the Orange Cliffs region (Shultz et al. 1987) and Capitol Reef National Park (Fertig 2009) and as common in the San Rafael Swell (Harris 1983).

#### *Streptanthella longirostris* (S.Watson) Rydb.

**Specimen Examined**: Utah, Emery County: *Sokoloff et al. 1261* (CAN).

This specimen is common in the MDRS area (Fig. 9d, e) and across the south-western United States (Al-Shehbaz 2010c). Phylogenetic work that includes this currently monotypic genus has shown that it is not monophyletic and that future taxonomic revision may be required (Ivalú Cacho et al. 2014). This species is present in the Orange Cliffs region (Shultz et al. 1987), widespread in Glen Canyon National Recreation Area (Hill and Ayers 2009), present in Capitol Reef National Park (Fertig 2009) and common in the San Rafael Swell (Harris 1983).

#### *Strigosella africana* (L.) Botsch.

**Specimens Examined**: Utah, Wayne County: *Sokoloff et al. 1269* (CAN, UTC); *1278* (CAN, UTC).

Commonly seen across the MDRS area (Fig. 9f), this invasive weed proliferates in disturbed areas across the western United States (Al-Shehbaz 2010b, Rollins 1980). This species has been reported as common in the San Rafael Swell (Harris 1983), present in Capitol Reef National Park (Fertig 2009) and as occasional in Glen Canyon Recreation Area (Hill and Ayers 2009). This species was recorded in these inventories as *Malcolmia africana* (L.) W.T. Aiton; however, it is now placed in *Strigosella* (Al-Shehbaz et al. 2014, Botschantzev 1972).

### Cactaceae

While conducting fieldwork at MDRS, Crew 210 occasionally encountered populations of small barrel cacti, consistent with members of the Cactaceae subfamily Cactoidae (Parfitt and Gibson 2003). Several species within this subfamily are known to occur within the San Rafael Swell and nearby Capitol Reef National Park (Fertig 2009, Harris 1983), at least two of which are listed as endangered species by the U.S. Federal Government (Fertig 2009). Therefore, we did not collect these species, but rather photo-documented these populations and examined them *in situ*. Though these populations were not yet flowering when examined, visible morphological characters were suffcient to conclude that these are likely populations of *Pediocactus* Britton & Rose and *Sclerocactus* Britton & Rose (Parfitt and Gibson 2003). Without specimens, we are hesitant to guess at species, but crews visiting the area should take care to avoid disrupting any cacti populations regardless of species identity or conservation status. To aid in conservation efforts, we are withholding the photos and exact localities of these populations.

In 2014, Crew 143 collected two cacti specimens (Sokoloff et al. 2016). Originally, they were identified as Opuntia basilaris var. basilaris Engelm. & J.M. Bigelow and Opuntia polyacantha var. polyacantha Haw.; however, these taxa have since been re-identified to Opuntia basilaris var. heilii S.L. Welsh & Neese and *Opuntia nicholii* L.D. Benson, respectively by *Opuntia* experts (Tony Frates and Dean Stock, personal communications, 2019).

### Caprifoliaceae

We documented one new species in one new genus for the MDRS area.

#### *Symphoricarpos longiflorus* Gray.

**Specimens Examined**: Utah, Wayne County: *Sokoloff et al. 1284* (CAN); *1296* (CAN, UTC).

This species was only encountered twice in the MDRS area (Fig. 10a), including several large plants in a sheltered shady wash with other locally-unique species, such as Populus fremontii var. fremontii and Rhus trilobata var. trilobata. This species is present in the Orange Cliffs region (Shultz et al. 1987), present in Capitol Reef National Park (Fertig 2009), occasional in the Glen Canyon National Recreation Area (Hill and Ayers 2009) and occasional in the San Rafael Swell (Harris 1983).

### Cleomaceae

We documented one new species in one new genus for the MDRS area.

#### *Cleomella palmeriana* M.E. Jones

**Specimen Examined**: Utah, Wayne County: *Sokoloff et al. 1311* (CAN, DAO, UTC).

Abundantly common on the silty floor of Copernicus Valley north of MDRS (Fig. 10b), where it was found growing with Phacelia demissa var. demissa, this annual plant is endemic to the Four Corners states (Vanderpool 2010). Infraspecific taxa are no longer recognised in this species following the transfer of C. palmeriana var. goodrichii Welsh to C. hillmanii var. goodrichii (S. L. Welsh) P.K. Holmgren (Holmgren 2004, Roalson et al. 2015), a move supported by genetic sequence data (Riser II et al. 2013). This species is common in Glen Canyon National Research Area (Hill and Ayers 2009), present in Capitol Reef National Park (as C. palmeriana var. palmeriana) (Fertig 2009) and occasional in the San Rafael Swell (Harris 1983).

### Fabaceae

We documented six new species in two new genera and one previously-documented genus for the MDRS area. We also made a collection of a species previously known from the station: *Astragalus desperatus* M.E. Jones (*Sokoloff et al. 1317b* [Fig. 11a, b]).

#### Astragalus mollissimus var. thompsoniae (S. Watson) Barneby

**Specimens Examined**: Utah, Wayne County: *Sokoloff et al. 1265* (CAN, UTC); *1299* (CAN, UTC).

Occasionally encountered in the MDRS area, this spring-flowering species was conspicuous in sandy washes (Fig. 11c, d). This variety is the only member of this widespread compound species present in Utah, where it is relatively common (Barneby 1964, Welsh et al. 1993, Welsh 2006). This taxon is present in the Orange Cliffs region (Shultz et al. 1987), present in Capitol Reef National Park (Fertig 2009), common in Glen Canyon National Recreation Area (Hill and Ayers 2009) and common in the San Rafael Swell (Harris 1983).

#### *Astragalus pardalinus* (Rydb.) Barneby

**Specimen Examined**: Utah, Wayne County: *Sokoloff et al. 1286* (CAN, UTC).

Given the non-flowering nature of this specimen, our identification here is tentatively made, based on vegetative morphology. This species was only encountered once in the MDRS area. This species is endemic to this region (Barneby 1964, Welsh et al. 1993, Welsh 2006) and, though not listed at the state or federal level, it is listed as a "Category C3 Non-Candidate" species in the "Watch" category of an inventory of Utah's sensitive plant life (Utah Division of Wildlife Resources 1998). This species is present in Capitol Reef National Park (Fertig 2009), occasional in Glen Canyon National Recreation Area (Hill and Ayers 2009) and occasional in the San Rafael Swell (Harris 1983).

#### *Astragalus praelongus* E. Sheld.

**Specimen Examined**: Utah, Wayne County: *Sokoloff et al. 1301* (CAN, UTC).

Only seen once along the side of a road northeast of MDRS, this large, showy milkvetch is apparently uncommon in the operational area (Fig. 11e). Barneby (1964) and Welsh (2006) largely agree on the infraspecific taxonomy of this species - Welsh describes a fourth variety from northwest Utah in addition to the three varieties present in both treatments. As our specimen was flowering, we have only identified it to species. This species is present in the Orange Cliffs region (Shultz et al. 1987), occasional in Glen Canyon National Recreation Area (Hill and Ayers 2009), present in Capitol Reef National Park (as var. praelongus) (Fertig 2009) and uncommon in the San Rafael Swell (as var. praelongus) (Harris 1983).

#### *Astragalus woodruffii* M.E. Jones

**Specimen Examined**: Utah, Wayne County: *Sokoloff et al. 1280* (CAN, DAO, UTC).

Occasional on sandy soils northeast of MDRS, this species is endemic to the San Rafael Swell and surrounding deserts (Barneby 1964, Welsh et al. 1993, Welsh 2006). As with *Astragalus pardalinus*, *A. woodruffii* is not state- or federally-listed, but is listed as category C3 Non-Candidate" species in the "Watch" category in Utah's sensitive plant life survey (Utah Division of Wildlife Resources 1998). This species is present in Capitol Reef National Park (Fertig 2009) and uncommon in the San Rafael Swell (Harris 1983).

#### *Hoffmannseggia repens* (Eastw.) Cockerell

**Specimen Examined**: Utah, Wayne County: *Sokoloff et al. 1280* (CAN, MT, UTC).

This species was occasionally encountered on sandy plains northeast of MDRS (Fig. 11f). Endemic to south-eastern Utah and adjacent Colorado (Simpson and Ulibarri 2006), this species is the northernmost member of a predominantly Central and South American genus (Simpson et al. 2004). This species is common in the San Rafael Swell (Harris 1983) and there are unconfirmed reports for Capitol Reef National Park (Fertig 2009).

#### *Lupinus pusillus* Pursh

**Specimen Examined**: Utah, Wayne County: *Sokoloff et al. 1287* (CAN, UTC).

This species was occasionally encountered on sandy plains northeast of MDRS (Fig. 12a). Welsh et al. (1993) describes three varieties present in Utah, but notes that, in his opinion, there was insufficient evidence to recognise them within the state. As our specimen is vegetative, we were unable to ascribe it to any variety. This species is present in the Orange Cliffs (Shultz et al. 1987), common in the San Rafael Swell (as *L. pusillus* var. pusillus and L. pusillus var. rubens (Rydb.) Welsh) (Harris 1983), present in Capitol Reef National Park (as L. pusillus var. pusillus and L. pusillus var. intermountanus (Heller) C.P. Sm.) (Fertig 2009) and present in Glen Canyon National Recreation Area (as L. pusillus subsp. pusillus and L. pusillus subsp. rubens (Rydb.) D.B. Dunn) (Hill and Ayers 2009).

### Loasaceae

We documented one new species in one new genus for the MDRS area.

#### *Mentzelia pterosperma* Eastw.

**Specimen Examined**: Utah, Wayne County: *Sokoloff et al. 1315* (CAN, UTC).

Only encountered once as a vegetative specimen on "Hab Ridge", on gravelly clay soil characteristic of its range in Utah (Schenk and Hufford 2016), this species may be more commonly encountered during flowering, when the plants are more conspicuous. This species is reported as occasional in Glen Canyon National Recreation Area (Hill and Ayers 2009), uncommon in the San Rafael Swell (Harris 1983) and present in Capitol Reef National Park (Fertig 2009).

### Nyctaginaceae

We documented one new species in one new genus for the MDRS area.

#### *Abronia elliptica* A. Nelson

**Specimen Examined**: Utah, Wayne County: *Sokoloff et al. 1292* (CAN, UTC).

The taxonomy of *A. fragrans* and *A. elliptica* A. Nelson has a complicated history in Utah, with Welsh et al. (1993) lumping both species in the state together, despite earlier (Galloway 1975) and later (Galloway 2003) treatments separating them based on fruiting characters. Here we follow Galloway (2003); however, the specimens we collected were vegetative (Fig. 12b). Pending collection of material with mature fruits, we tentatively assign this specimen to *A. elliptica*, as continuing work by Eric LoPresti indicates that the majority of specimens from this complex in cental Utah properly belong in this species, particularly around the San Rafael Swell (Mike Moore, personal communication, 2020). *Abronia elliptica* is reported as common in the San Rafael Swell (Harris 1983) and present in the Orange Cliffs Region (Shultz et al. 1987), while *A. fragrans* is not recorded at all. Conversely, *A. fragrans* (with *A. elliptica* in synonymy) is reported as present in Capitol Reef National Park (Fertig 2009) and widespread in Glen Canyon National Recreation Area (Hill and Ayers 2009). Further work will be needed to verify the true range of this species in the regions surrounding MDRS.

### Onagraceae

We documented two new species in one new genus and one previously-documented genus for the MDRS area. We also made a collection of a species previously known from the station: Oenothera cespitosa subsp. navajoensis W.L. Wagner, Stockhouse & Klein M.E. Jones (*Sokoloff et al. 1279*).

#### *Camissonia eastwoodiae* (Munz) P.H. Raven

**Specimens Examined**: Utah, Wayne County: *Sokoloff et al. 1307* (CAN); *1313* (CAN, UTC).

A Colorado Plateau endemic (Welsh et al. 1993), this species was encountered occasionally around MDRS on the clay sediments in Copernicus Valley and on grey Mancos Shale layers (Fig. 12c), consistent with published habitat descriptions for this species (Raven 1969). Reported as common in the San Rafael Swell (Harris 1983), present in Capitol Reef National Park (Fertig 2009) and common in Glen Canyon National Recreation Area (Hill and Ayers 2009).

#### *Oenothera pallida* Lindl.

**Specimen Examined**: Utah, Wayne County: *Sokoloff et al. 1288* (CAN).

This species was only encountered once in the MDRS area as an inconspicuous vegetative specimen; fieldwork during this species' flowering time may reveal it to be common in the area. The taxonomy of this group has fluctuated, with Welsh et al. (1993) recognising two varieties in Utah and Evans et al. (2005) recognising five subspecies across this species range. As the specimen cited here is immature, we have only determined it to the species level. This species has been reported from the Orange Cliffs (Shultz et al. 1987), as widespread in the Glen Canyon National Recreation Area (Hill and Ayers 2009), present in Capitol Reef National Park (Fertig 2009) and as common in the San Rafael Swell (Harris 1983).

### Poaceae

We documented two new species in two new genera the MDRS area. We also made collections of species previously known from the station, including: *Achnatherum hymenoides* (Roem. & Schult.) Barkworth (*Sokoloff et al. 1282*) and *Sporobolus airoides* (Torr.) Torr. (*Sokoloff et al. 1294*).

#### *Eremopyrum triticeum* (Gaertn.) Nevski

**Specimen Examined**: Utah, Wayne County: *Sokoloff et al. 1321* (CAN, UTC).

Though not reported from the San Rafael Swell (Harris 1983), Capitol Reef National Park (Fertig 2009) or Glen Canyon National Recreation Area (Hill and Ayers 2009), Frederiksen (2007) reports this annual weed as occurring across western North America, scattered across disturbed sites and Barkworth et al. (1983) reports this species as occurring across the Intermountain Region. At MDRS, this species was only found in areas immediately adjacent to the station (Fig. 12d, e), in disturbed, saline clay sediment matching the habitat description in Banner et al. (2011).

#### Vulpia octoflora (Walter) Rydb. var. octoflora

**Specimens Examined**: Utah, Wayne County: *Sokoloff et al. 1274* (CAN, UTC); *1295* (CAN, UTC).

**Figure 13.**
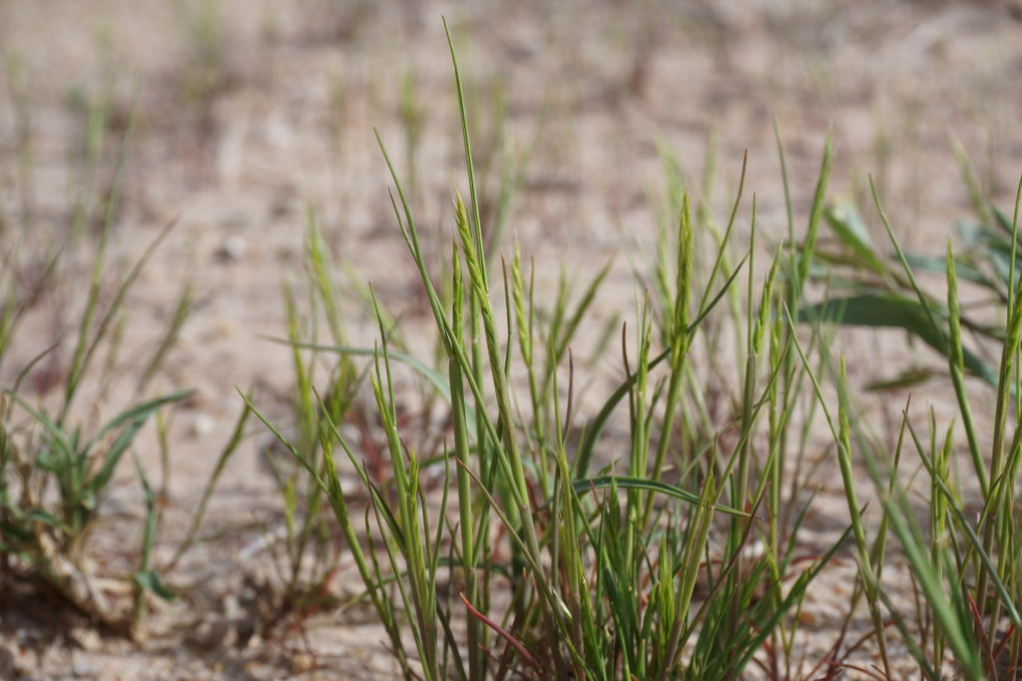
Vulpia octoflora var. octoflora, habit (*Sokoloff et al. 1274*). Photo by P.C. Sokoloff.

Widespread across North America (Lonard 2007), this annual taxon was commonly encountered across the MDRS area during our study (Fig. 13). This genus is sometimes placed in *Festuca* (Lonard and Gould 1974). As *Festuca octoflora* Walter, this species is reported from Capitol Reef National Park (Fertig 2009). As *Vulpia octoflora*, this species is reported as widespread in Glen Canyon National Recreation Area (Hill and Ayers 2009), present in the Orange Cliffs region (Shultz et al. 1987) and occasional in the San Rafael Swell (Harris 1983).

### Polygonaceae

We documented two new species in one known genus for the MDRS area.

#### *Eriogonum gordonii* Benth.

**Specimen Examined**: Utah, Wayne County: *Sokoloff et al. 1305* (CAN, UTC).

Common in the Four Corners states, Wyoming, South Dakota and Nebraska (Reveal 2005), this species was only encountered once in the MDRS area. This species is present in Capitol Reef National Park (Fertig 2009) and uncommon in the San Rafael Swell (Harris 1983).

#### *Eriogonum wetherillii* Eastw.

**Specimens Examined**: Utah, Wayen County: *Sokoloff et al. 1270* (CAN); *1272* (CAN, UTC).

Encountered occasionally in the MDRS area on sandy washes and clay soils (Fig. 12f), this species is endemic to the Four Corners states and common in southeast Utah (Reveal 2005). This species is present in the Orange Cliffs Region (Shultz et al. 1987), common in Glen Canyon National Recreation Area (Hill and Ayers 2009), present in Capitol Reef National Park (Fertig 2009) and occasional in the San Rafael Swell (Harris 1983).

### Salicaceae

We documented one new species in one new genus for the MDRS area.

#### Populus fremontii S. Watson subsp. fremontii

**Specimen Examined**: Utah, Wayne County: *Sokoloff et al. 1300* (CAN, UTC).

**Figure 14.**
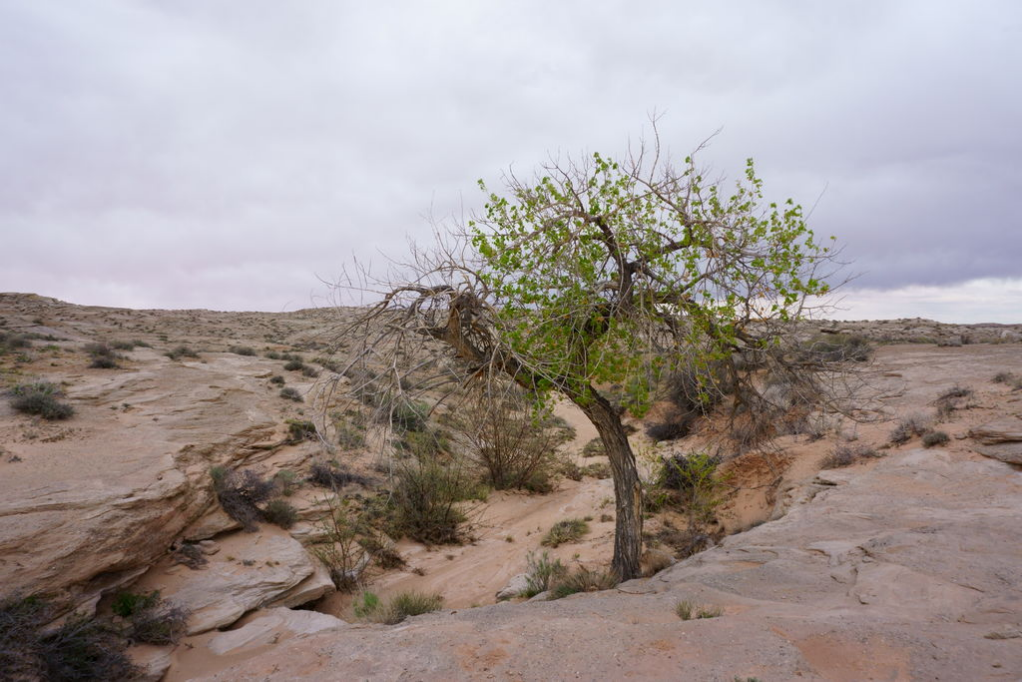
Populus fremontii subsp. fremontii, habit and habitat (*Sokoloff et al. 1300*). Photo by P.C. Sokoloff.

This species is common along the banks of the Fremont River south of the MDRS area, just south of Utah State Route 24 (P. Sokoloff, pers. obs.). This particular collection is the nearest-known population to MDRS and the only one encountered in the operational area in many years of fieldwork (S. Rupert, pers. obs). Consisting of one large tree and two smaller saplings (Fig. 14), this population was found in a protected sandy wash with other unique species in the MDRS area, including Rhus trilobata var. trilobata and *Symphoriocarpos longiflorus*. This taxon, recorded as *Populus fremontii*, has been reported as common in the San Rafael Swell (Harris 1983), occasional in the Glen Canyon National Recreation Area (Hill and Ayers 2009) and present in the Orange Cliffs region (Shultz et al. 1987). This taxon has also been reported as present in Capitol Reef National Park as Populus fremontii var. fremontii (Fertig 2009). Following Eckenwalder (2010), these plants are all part of the more widely distributed nominate subspecies; the other subspecies: P. fremontii subsp. mesetae Eckenwalder, is only found in Texas.

## Discussion

Adding our current inventory to the vascular plant list in our earlier work (Sokoloff et al. 2016) brings the total number of vascular plant taxa at MDRS to 81 (79 species and two taxa recorded to genus) in 24 familes; our current study represents a ~110% increase in species diversity from our initial survey. As we expected, work in the spring greatly increased the number of taxa encountered, and working outside of Mars simulation conditions with a crew of dedicated biologists resulted in Crew 210 doubling the species list for MDRS in half the time that Crew 143 spent at the station.

Nine new families are documented for the MDRS area (Amaryillidacaeae, Anacardiaceae, Apiaceae, Asparagaceae, Caprifoliacaeae, Cleomaceae, Loasaceae, Nyctinaginaceae and Salicaceae). The remaining nine families that contain new species for the MDRS area (Amaranthaceae, Asteraceae, Boraginacaeae, Brassicaceae, Cactaceae, Fabaceae, Onagraceae, Poaceae and Polygonaceae) are relatively species-rich in the deserts of southeast Utah (Fertig 2009, Harris 1983) and were previously documented in the area (Sokoloff et al. 2016). Six families from our initial inventory are not represented in the new collections reported here (Ephedraceae, Euphorbiaceae, Juncaceae, Malvaceae, Sarcobatacaeae and Tamaricaceae). Of these families, Ephedraceae, Sarcobataceae and Tamaricaceae are large, conspicuous shrubby families with 1-2 species in the area (Fertig 2009, Harris 1983), all of which were observed, but not collected in 2019. New species in the Euphorbiaceae, Juncaceae and Malvaceae were not encountered in 2019.

Species were scored as annual or perennial based on the USDA PLANTS Database (USDA, NRCS 2020) - any species that may occur annually were scored as such. Overall, 43% of our new vascular plant records are annuals: Phacelia demissa var. demissa, *Cleomella palmeriana*, *Eremopyrum triticeum*, Cryptantha crassisepala var. elachantha, Vulpia octoflora var. octoflora, Lupinus pusillus, *Camissonia eastwoodiae*, *Chorispora tenella*, Descurainia pinnata subsp. brachycarpa, Streptanthella longirostris, Strigosella africana, Eriogonum gordonii, Eriogonum wetherillii, Atriplex argentea, Blitum nuttallianum, Phacelia corrugata, Chaenactis stevioides and *Malacothrix sonchoides.* This is much higher than the 29% of annual species from our 2014 inventory. Two of the taxa recorded here are geophytes: *Allium macropetalum* and *Eremocrinum albomarginatum* (as recorded in Flora of North America Editorial Committee, eds. (1993)), which would likewise not have been conspicuous during our earlier fall fieldwork. Additionally, *Pediocactus* is known to retract below ground when conditions are unfavourable (Shryock et al. 2014), reducing the likelihood that this species would be encountered in autumn or in drought years. Our 2019 fieldwork took place during a high-productivity year in the Utah desert (pers. obs.).

The San Rafael Swell and its surrounding deserts are habitat to numerous endemic vascular plant species (Harris 1983, Welsh et al. 1993, Flora of North America Editorial Committee, eds. 1993); our crew documented several occurrences of these species around MDRS, including *Hoffmannseggia repens*, *Astragalus pardalinus*, *Astragalus woodruffii* and populations of *Sclerocactus* and *Pediocactus*. We also documented populations of species endemic to the wider Colorado Plateau, like *Eremocrinum albomarginatum*, *Camissonia eastwoodiae* and Phacelia demissa var. demissa. These new records not only document the occurrence of these species at MDRS for future crews and biodiversity researchers, but provide important spatial and temporal records of these unique species to protected area managers and conservation planners.

Three of the species newly recorded for MDRS are invasive weeds in the southwest United States: *Eremopyrum triticeum*, *Strigosella africana* and *Chorispora tenella* (we also made one collection of the previously-documented weed *Kali tragus*). Documenting the occurrence of these taxa through vouchered herbarium specimens will provide important information to land managers working to control these invasive species.

While we have greatly increased the number of vascular plant species known at MDRS, the high diversity documented for the nearby San Rafael Swell and Capitol Reef National Park indicate that there are many species not yet documented for the station. Filling in the gaps in this checklist will require additional field seasons in spring, summer and early autumn and continued botanical exploration of both previously-inventoried and newly-documented sites within the MDRS area. Consulting specimens from local herbaria (and those served in online databases) may provide species occurrence data from the regions around MDRS helpful in the search for new station taxa. Additionally, crews rotating though MDRS may add to the inventory effort through photo-documenting vascular plant species encountered and uploading these images to online databases like iNaturalist.

Continuing to collect and add to the ongoing floristic inventory of MDRS will greatly aid future missions at the station where crews need a local taxonomic checklist (for environmental DNA studies, for example), but also highlights the importance of an expanded mission profile at MDRS. As our knowledge of the local biota at the station increases, this unique place can become a hub for earthbound biodiversity monitoring, in addition to its important role in Martian analogue research.

## Supplementary Material

6D057EE5-EFE1-5AC5-A13A-4985E2966155 10.3897/BDJ.8.e55063.suppl1 Supplementary material 1 Crew 210 Collections Data type occurences File: oo_416343.xlsx https://binary.pensoft.net/file/416343 P.C. Sokoloff; S.R. McBeth, D.A. Murray, S.M. Rupert, M.G. Irvine

## Figures and Tables

**Figure 1a.**
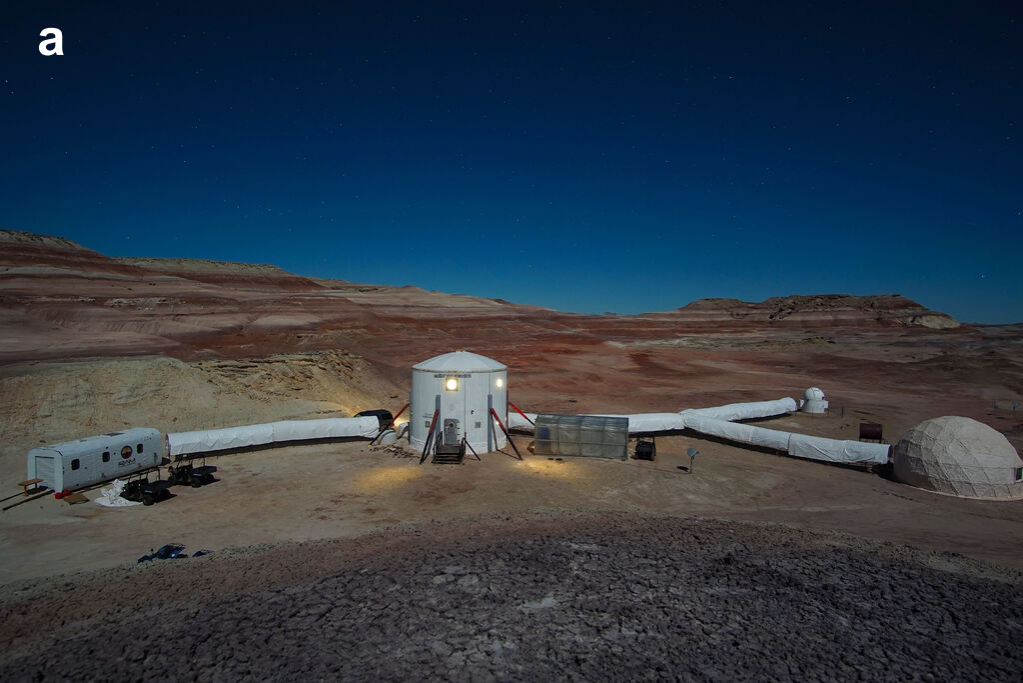
MDRS campus (L to R), the Repair and Maintenance Module (RAMM), the Hab, the GreenHab, the Robotic Observatory and the Science Dome.

**Figure 1b.**
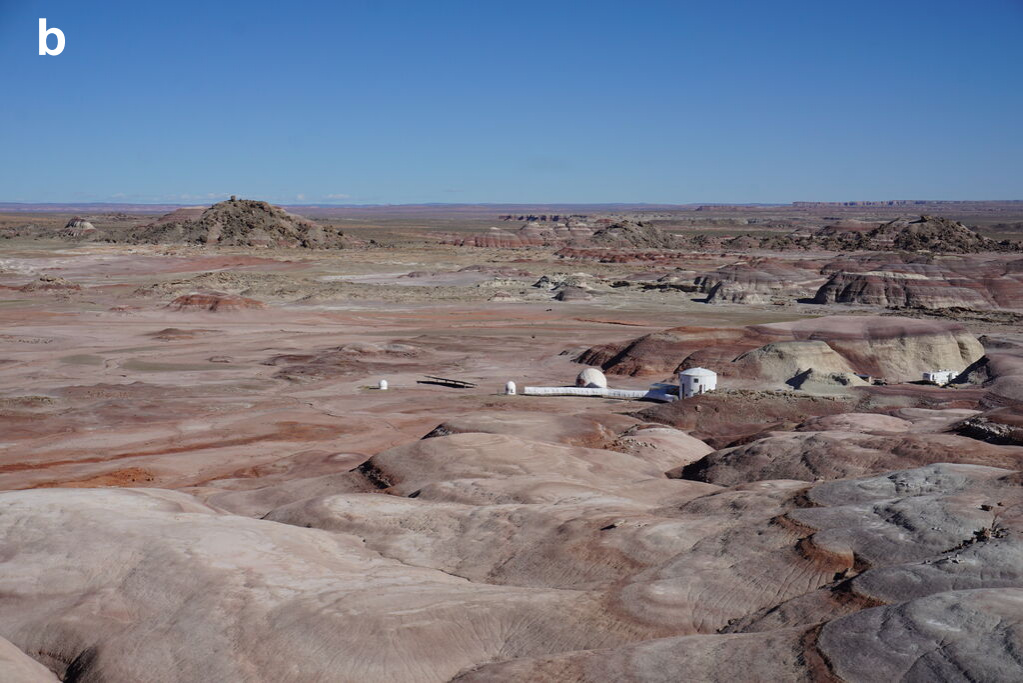
The analogue environment surrounding MDRS. "Hab Ridge" rises immediately to the right of MDRS and "Phobos Peak" is the hill in the left background.

**Figure 3a.**
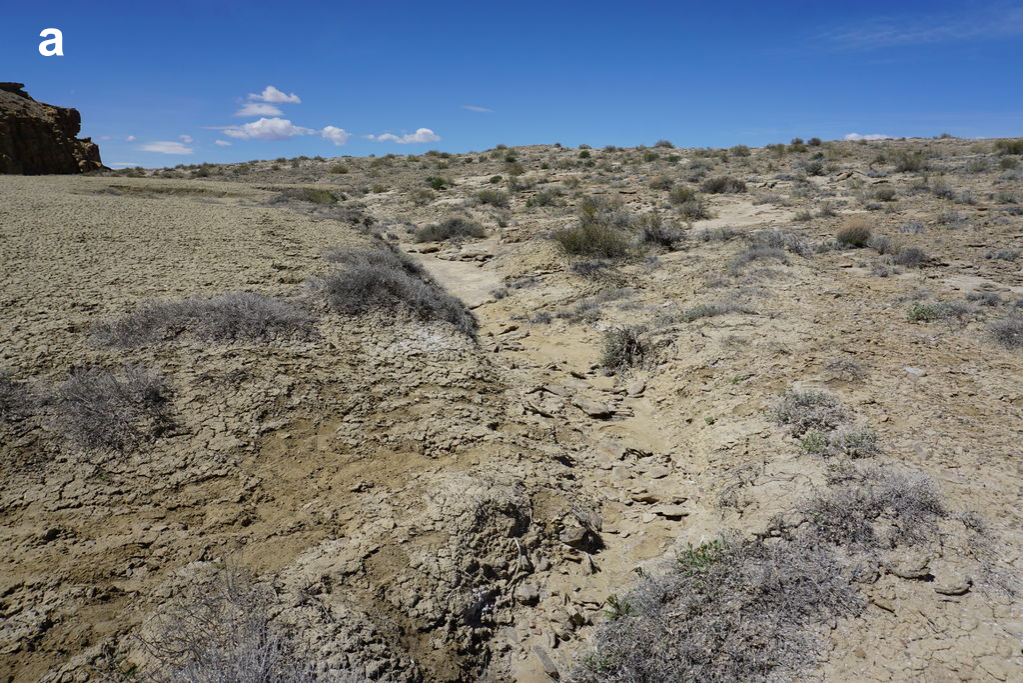
Valley of the Stars, Emery County (*Sokoloff et al. 1260-1264*).

**Figure 3b.**
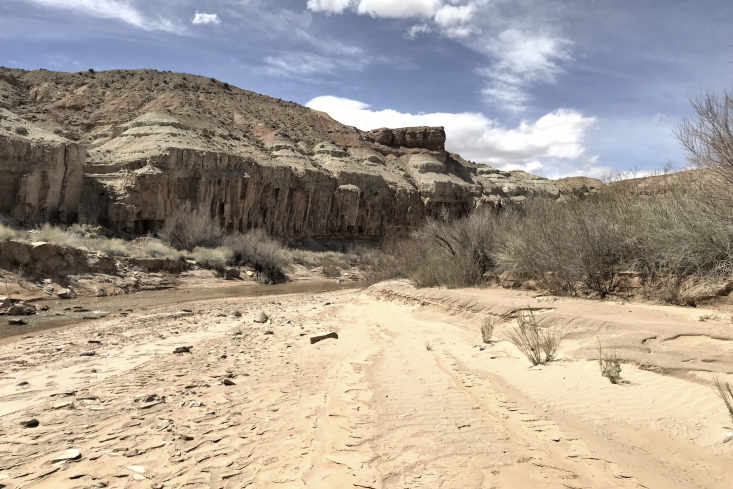
Salt Wash, Wayne County (*Sokoloff et al. 1265-1270*).

**Figure 3c.**
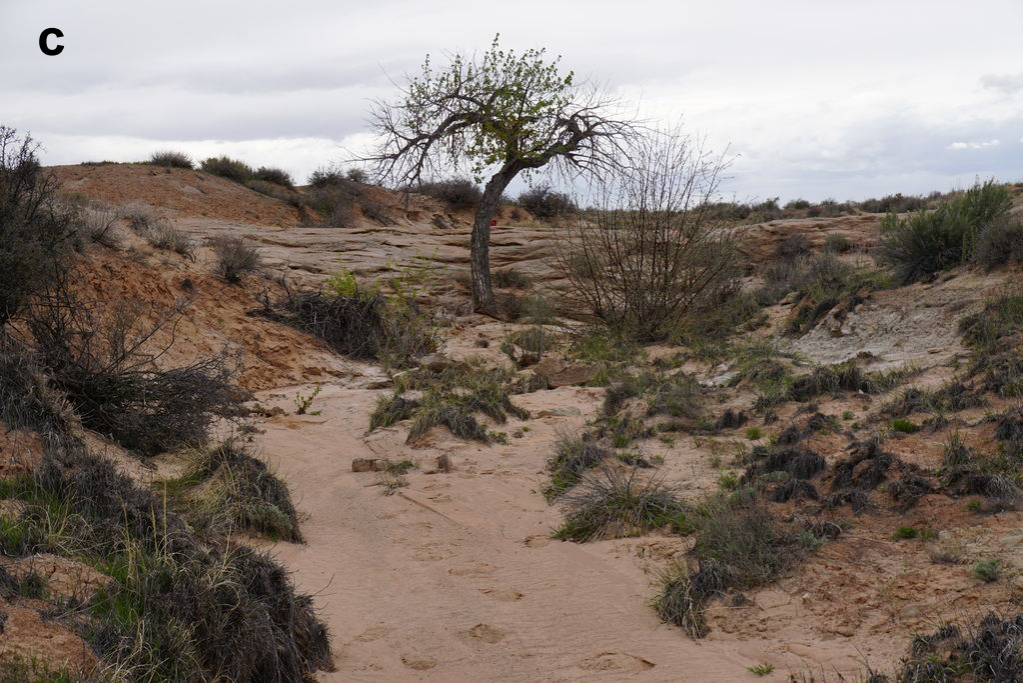
Sandy wash, Wayne County (*Sokoloff et al. 1296-1300*).

**Figure 3d.**
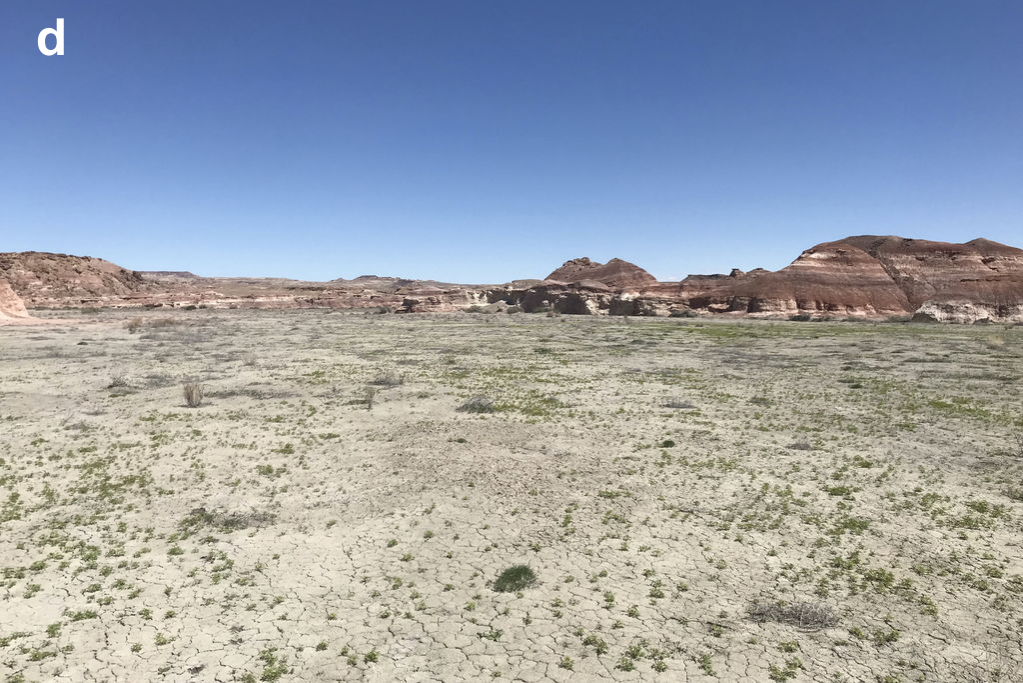
"Copernicus Valley", Wayne County (*Sokoloff et al. 1310-1314*).

**Figure 3e.**
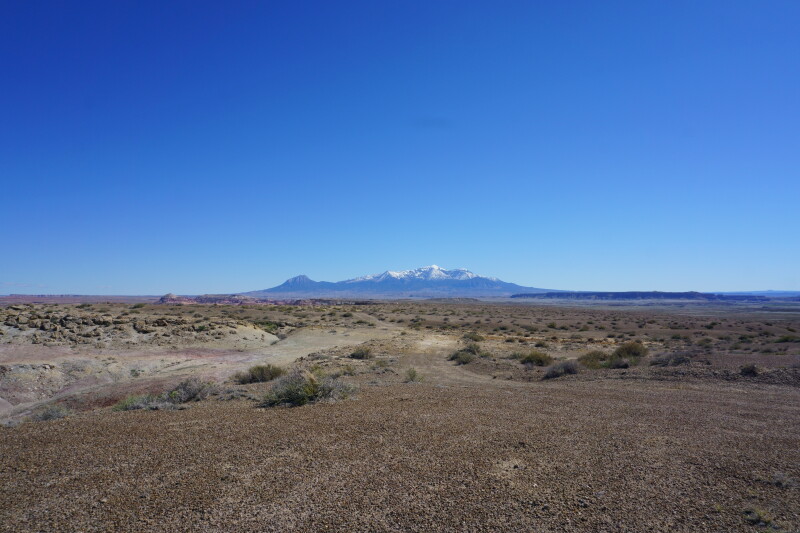
"Hab Ridge", Wayne County (*Sokoloff et al. 1315-1317*).

**Figure 3f.**
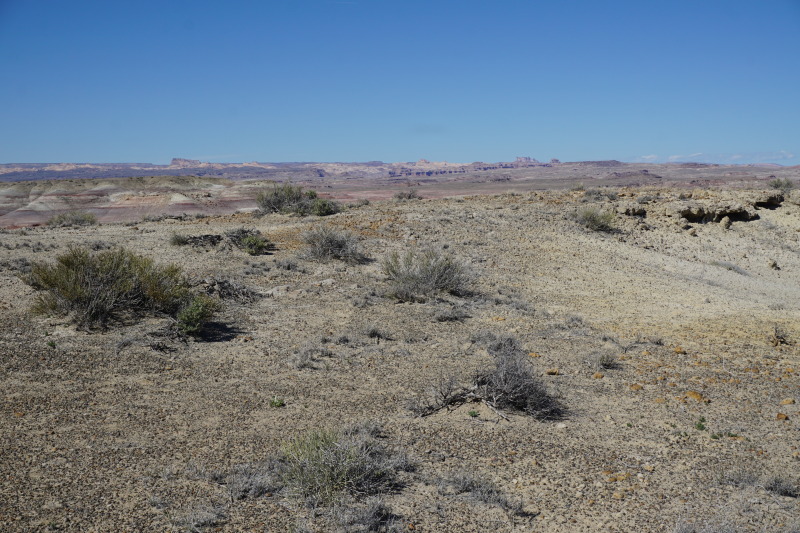
"Hab Ridge", Wayne County (*Sokoloff et al. 1315-1317*).

**Figure 4a.**
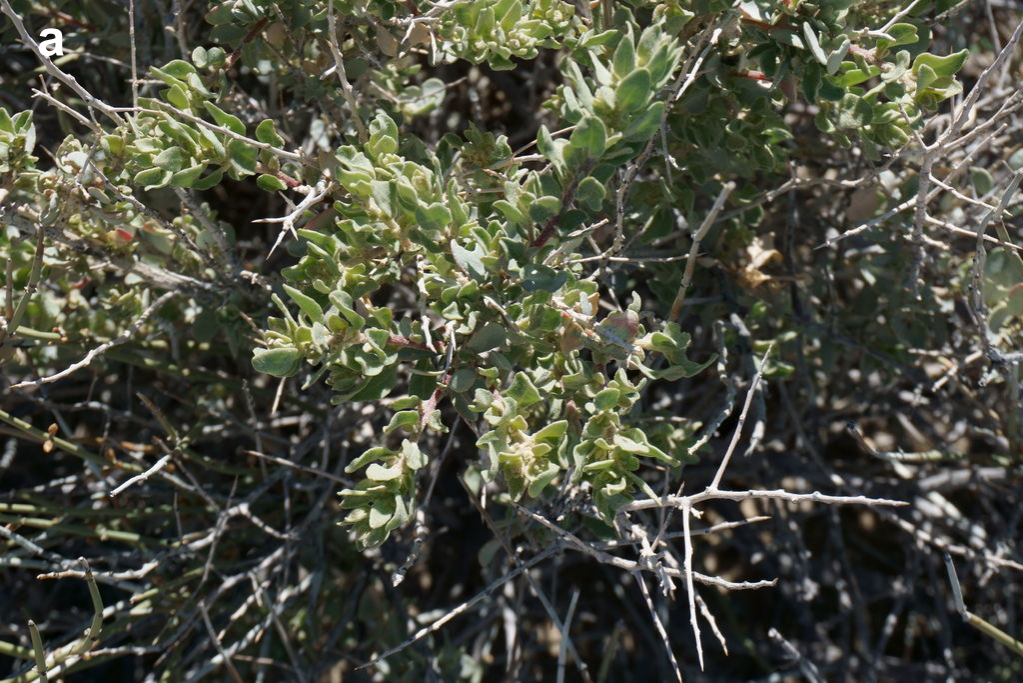
*Atriplex confertifolia*, branches and infructescence (*Sokoloff et al. 1317*).

**Figure 4b.**
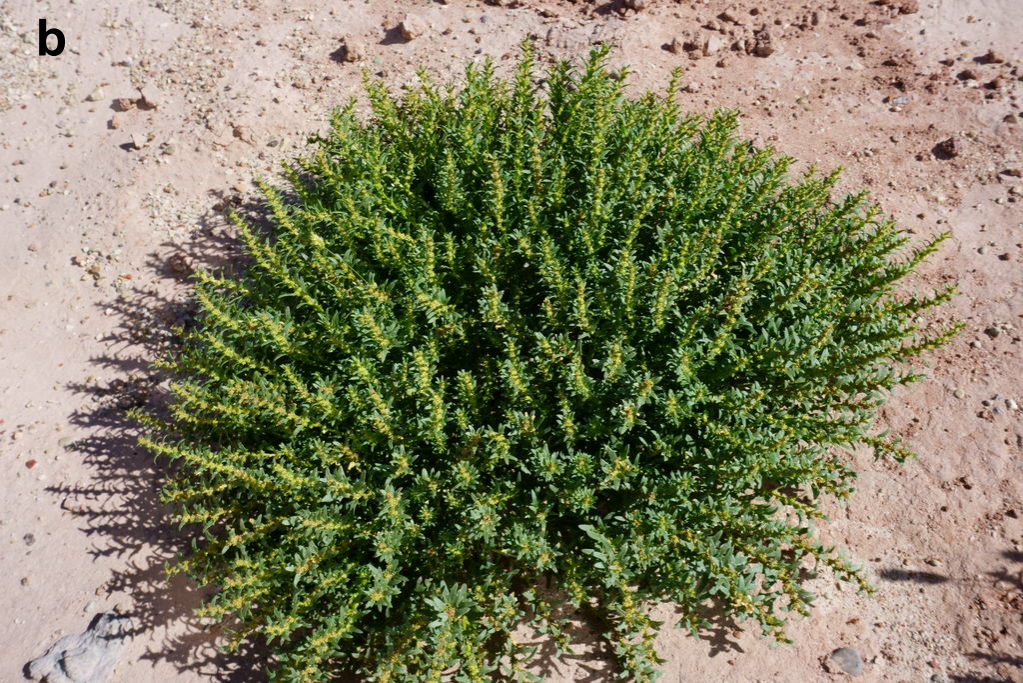
*Blitum nuttallianum*, habit (*Sokoloff et al. 1320*).

**Figure 4c.**
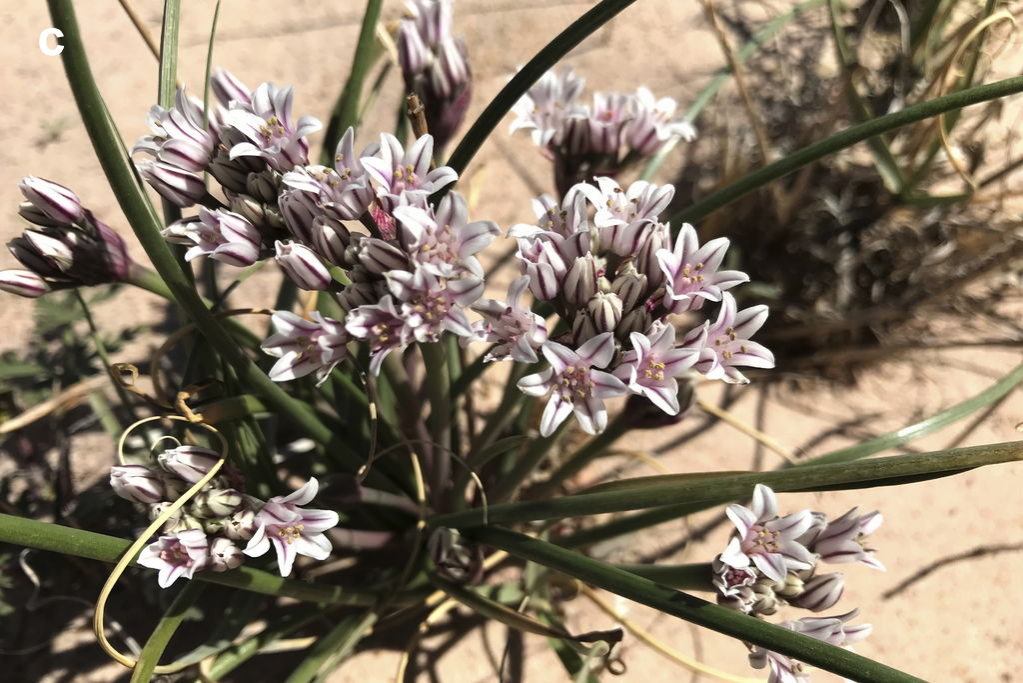
*Allium macropetalum* (*Sokoloff et al. 1319*).

**Figure 4d.**
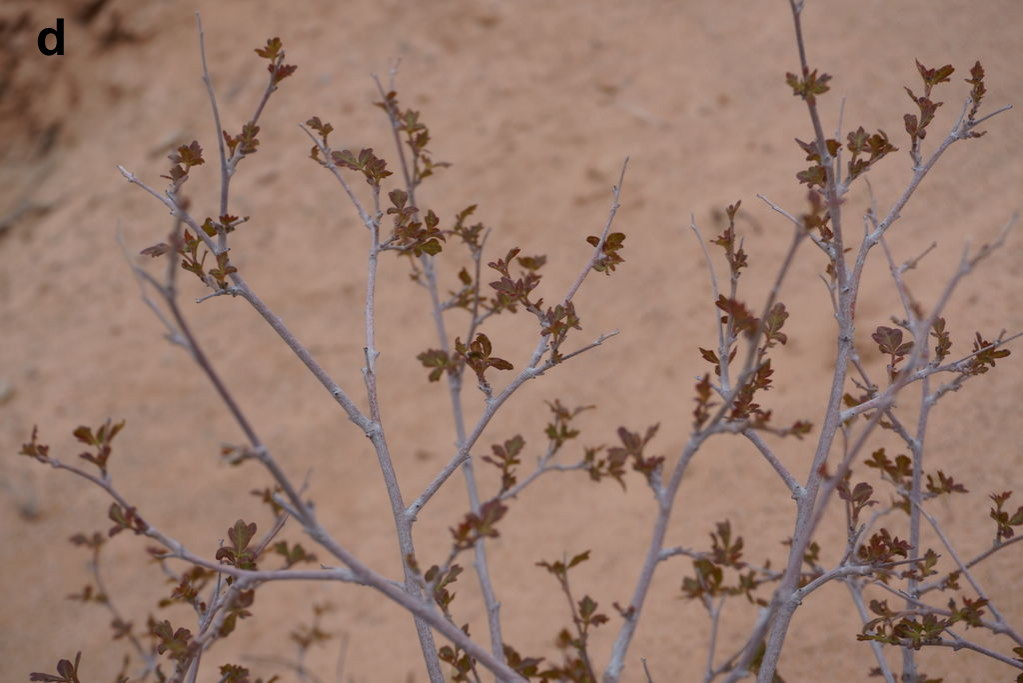
Rhus trilobata var. trilobata, leaf detail (*Sokoloff et al. 1297*).

**Figure 4e.**
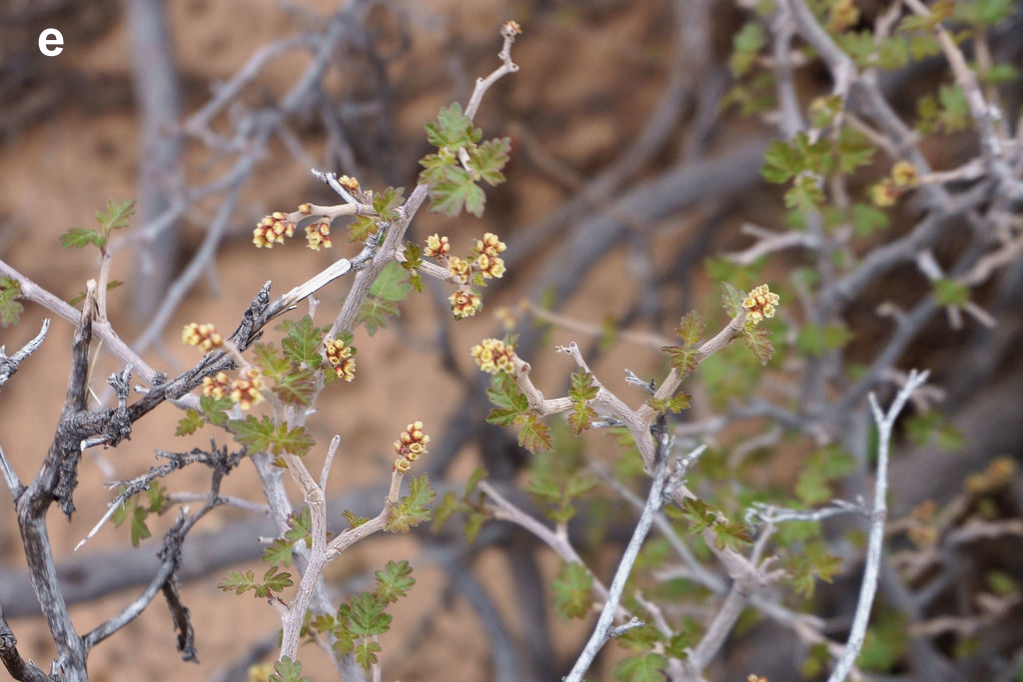
Rhus trilobata var. trilobata, inflorescences (*Sokoloff et al. 1297*).

**Figure 4f.**
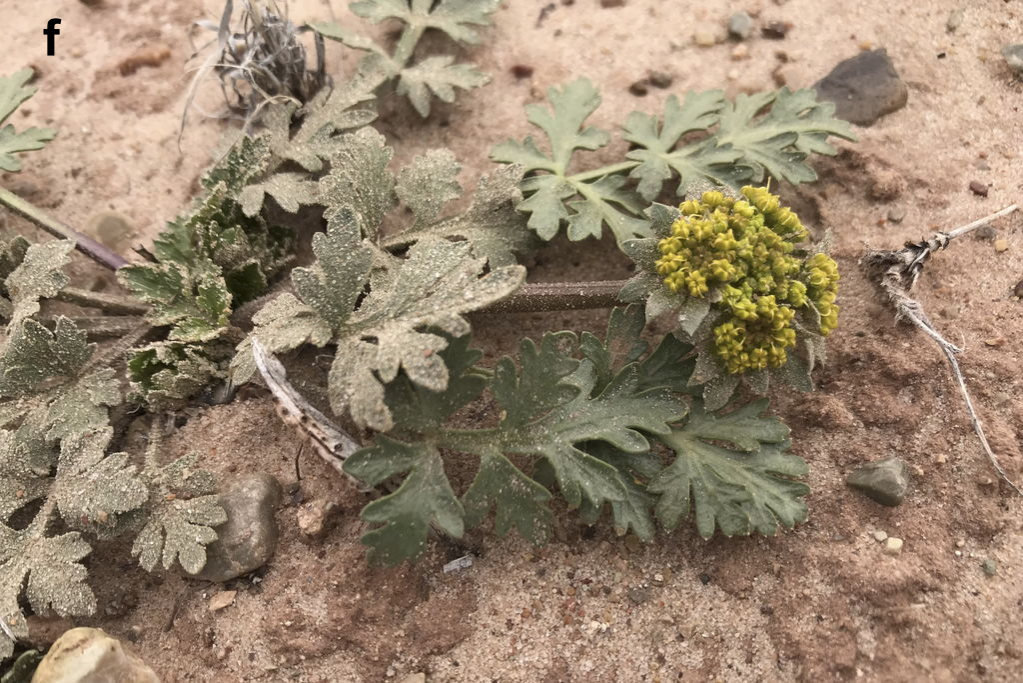
*Cymopterus glomeratus*, inflorescence (*Sokoloff et al. 1273*).

**Figure 5a.**
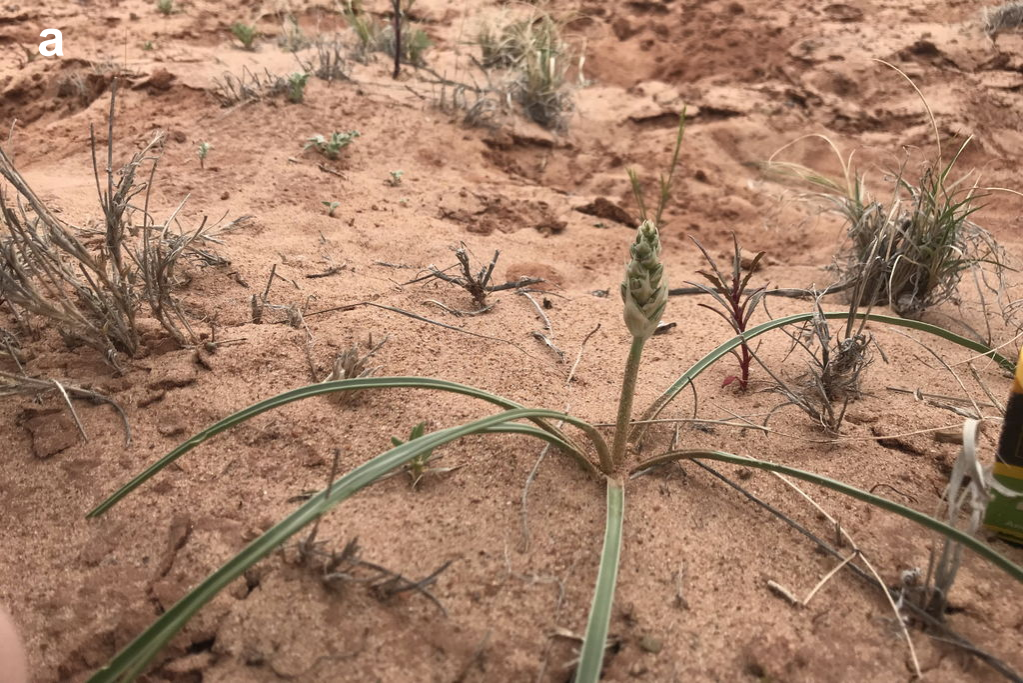
*Eremocrinum albomarginatum*, habit (*Sokoloff et al. 1289*).

**Figure 5b.**
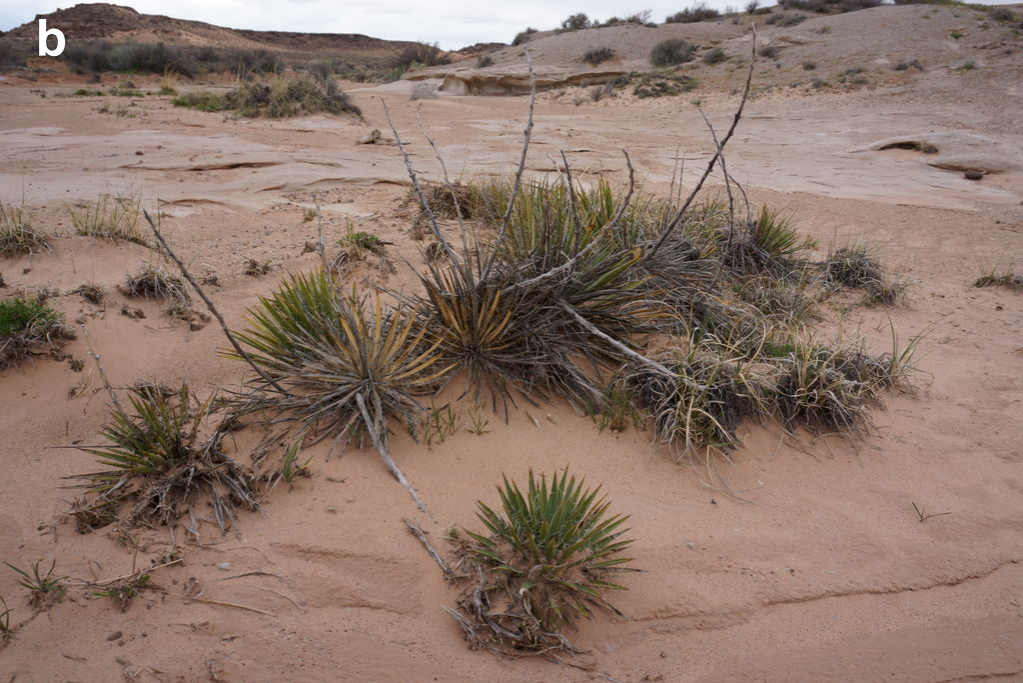
*Yucca harrimaniae*, habitat (*Sokoloff et al. 1298*).

**Figure 5c.**
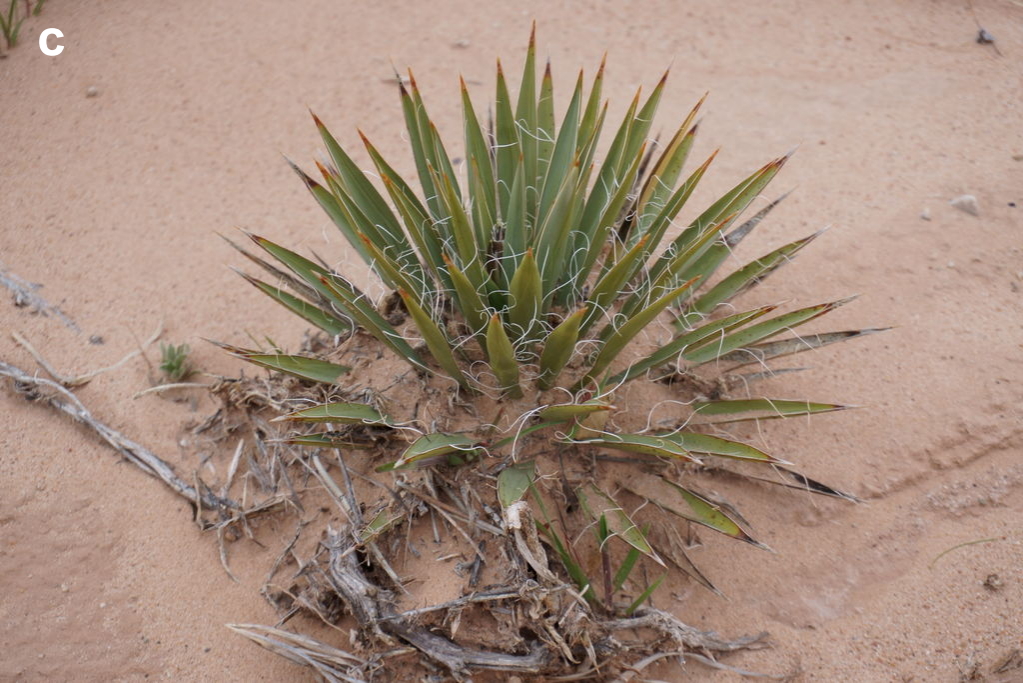
*Yucca harrimaniae*, habit (*Sokoloff et al. 1298*).

**Figure 5d.**
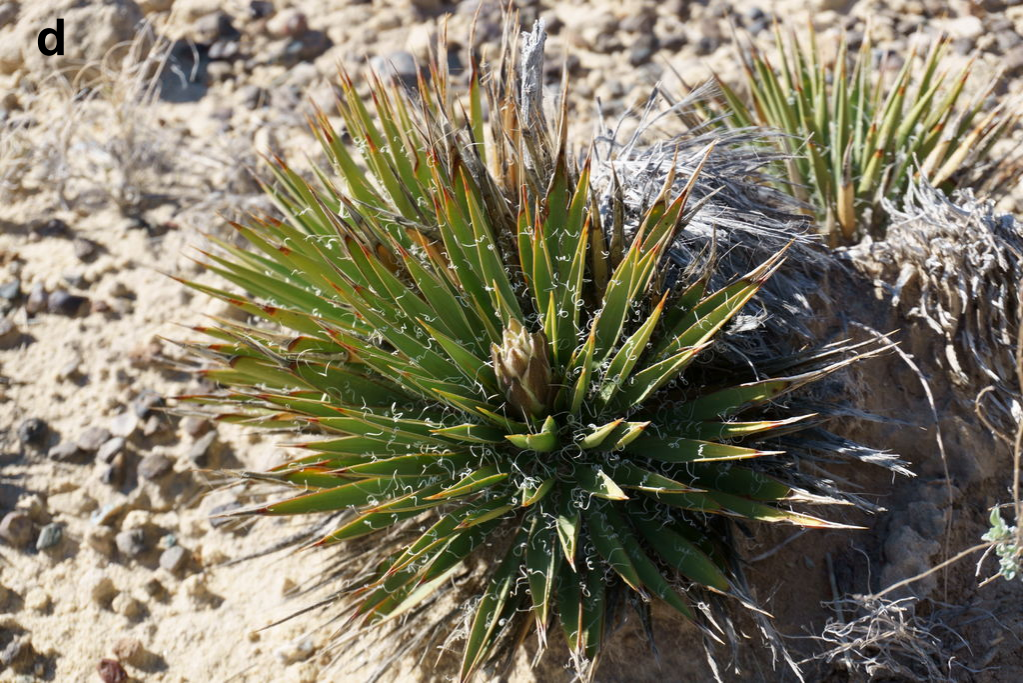
*Yucca harrimaniae*, developing inflorescence ("Hab Ridge" west of MDRS).

**Figure 6a.**
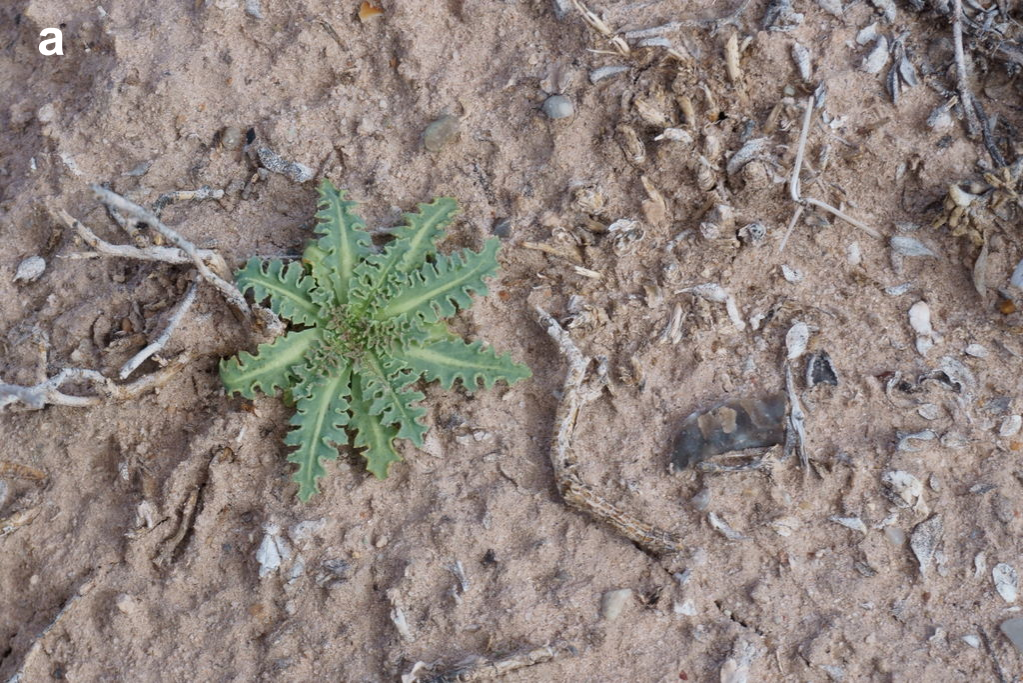
*Malacothrix sonchoides*, basal rosette (*Sokoloff et al. 1277*).

**Figure 6b.**
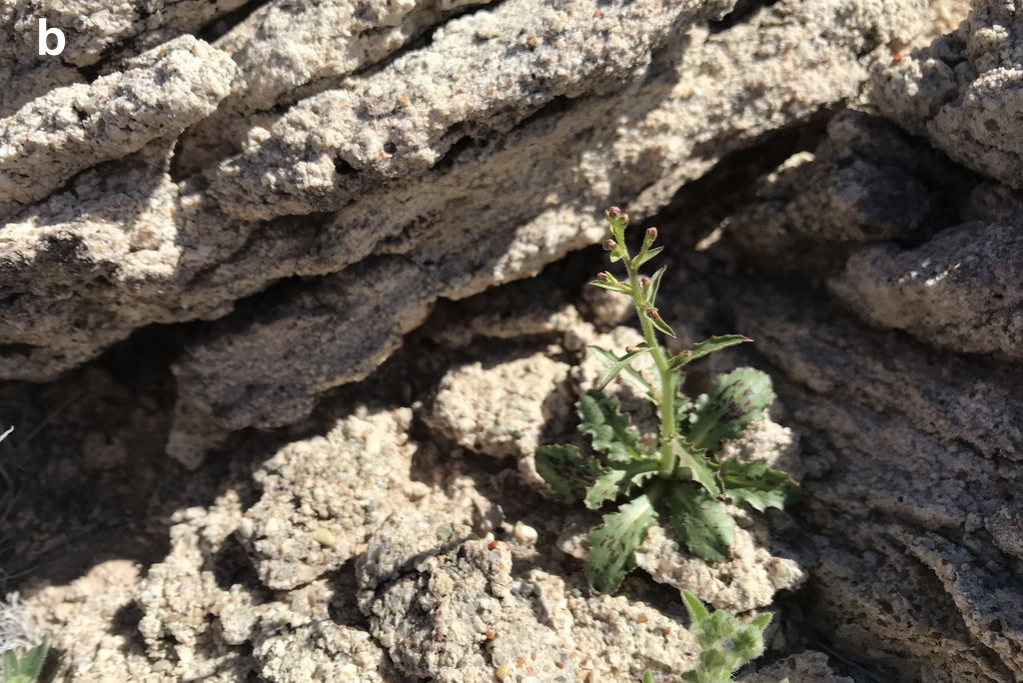
*Prenanthella exigua*, habit (*Sokoloff et al. 1306*).

**Figure 6c.**
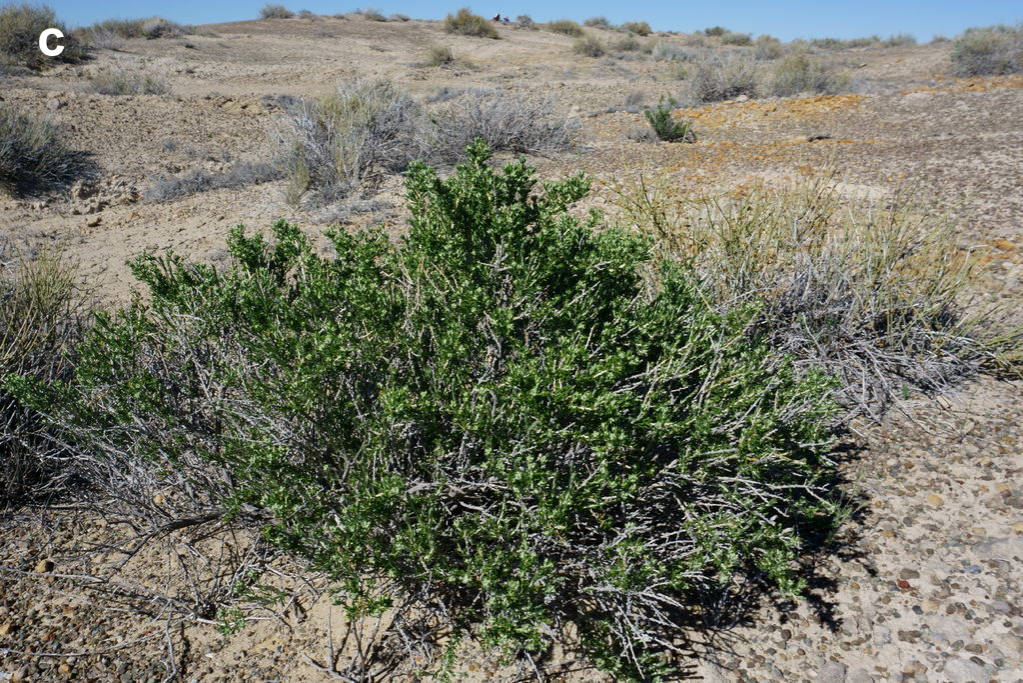
*Tetradymia glabrata*, habit (*Sokoloff et al. 1316*).

**Figure 6d.**
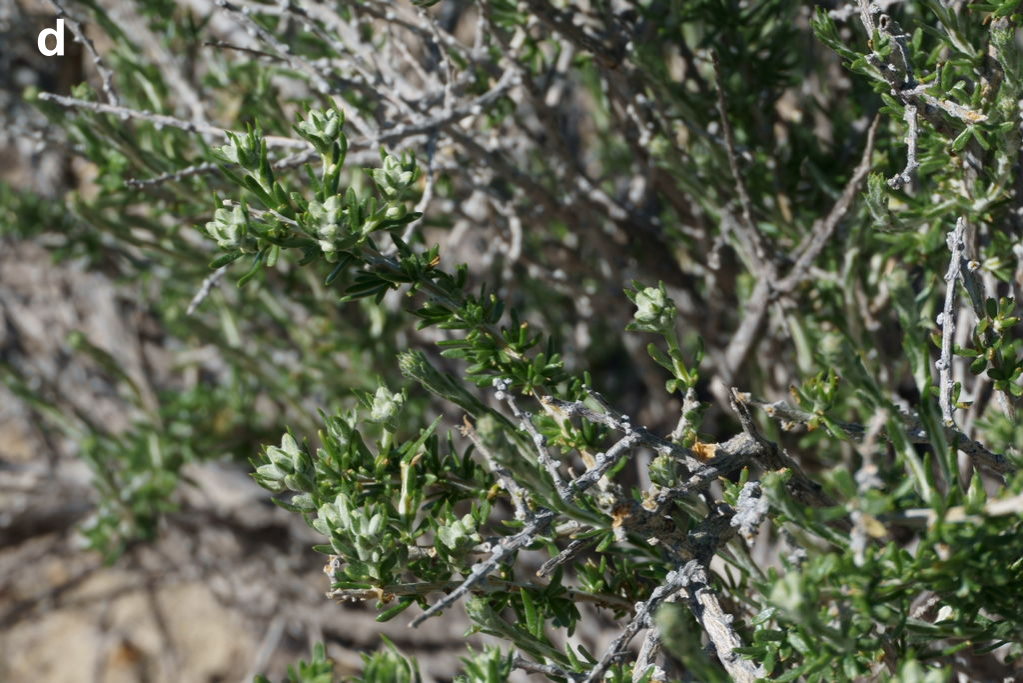
*Tetradymia glabrata*, leaf detail (*Sokoloff et al. 1316*).

**Figure 7a.**
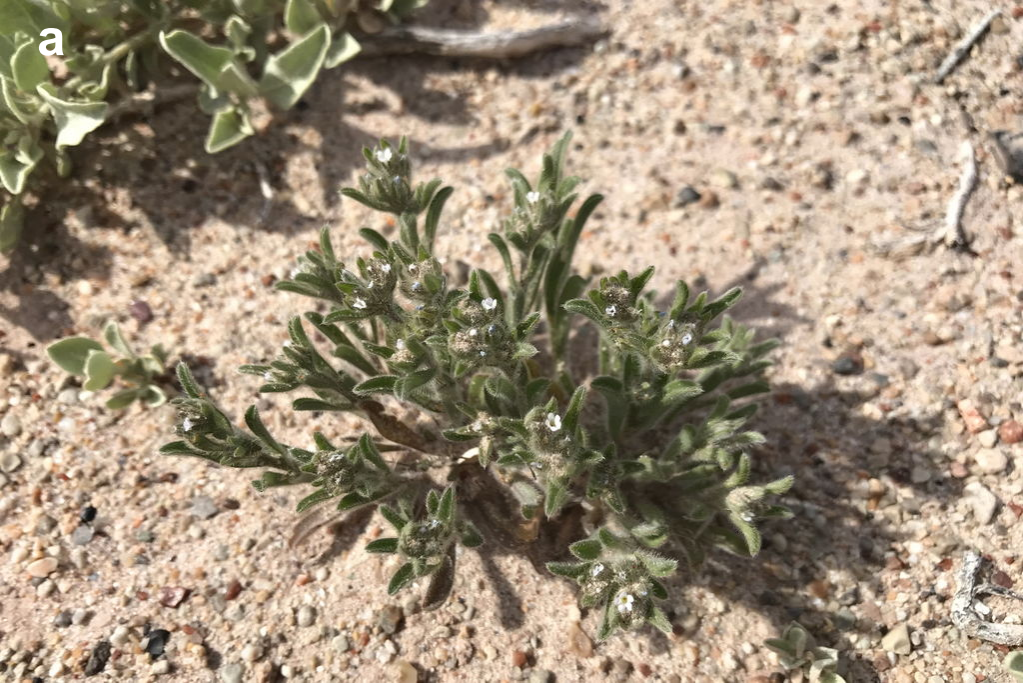
Cryptantha crassisepala var. elachantha, habit (*Sokoloff et al. 1276*).

**Figure 7b.**
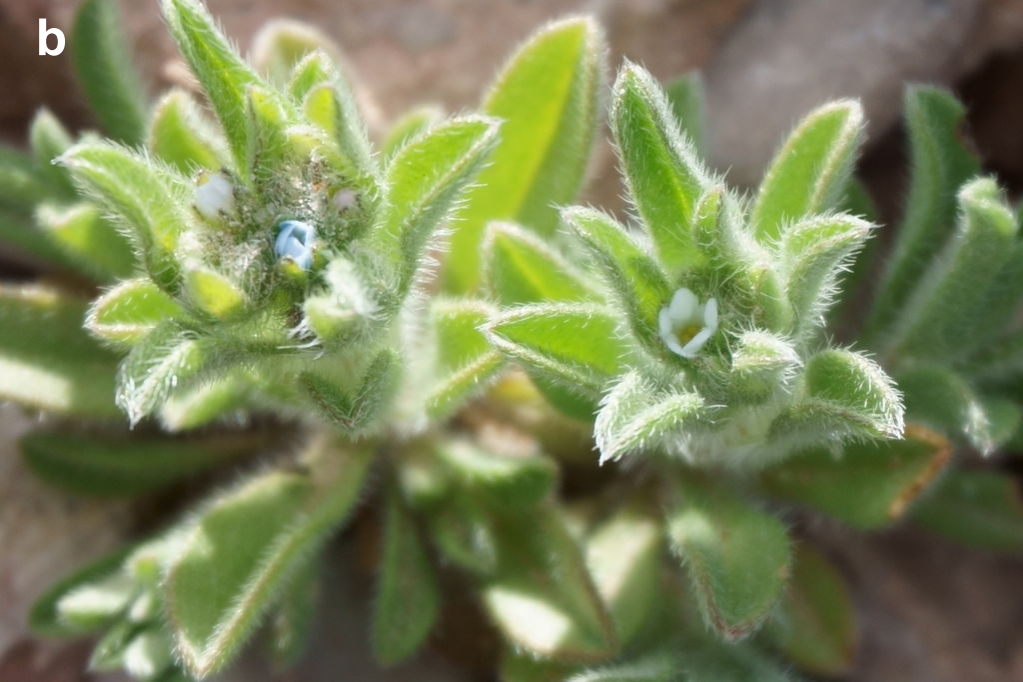
Cryptantha crassisepala var. elachantha, inflorescence (*Sokoloff et al. 1276*).

**Figure 7c.**
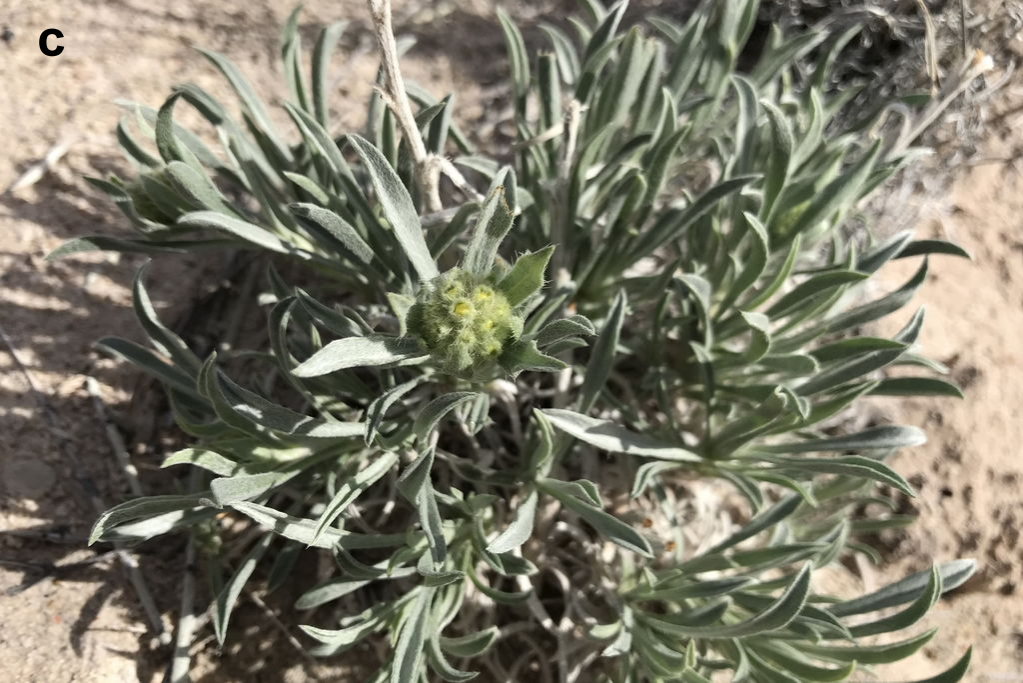
*Oreocarya flava*, habit (*Sokoloff et al. 1275*).

**Figure 7d.**
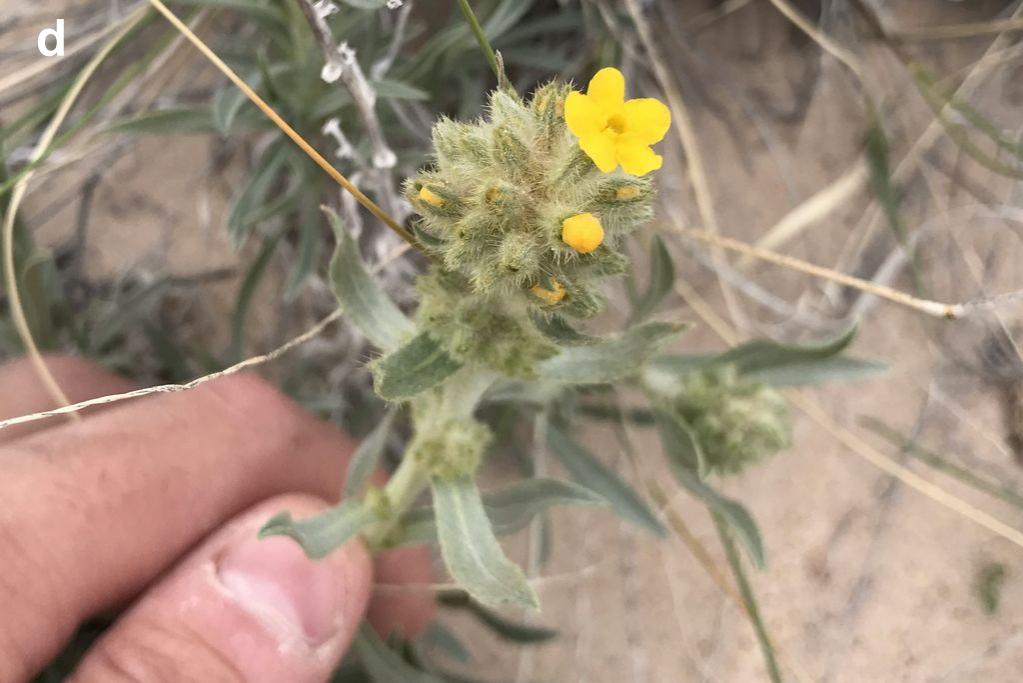
*Oreocarya flava*, inflorescence (*Sokoloff et al. 1291*).

**Figure 7e.**
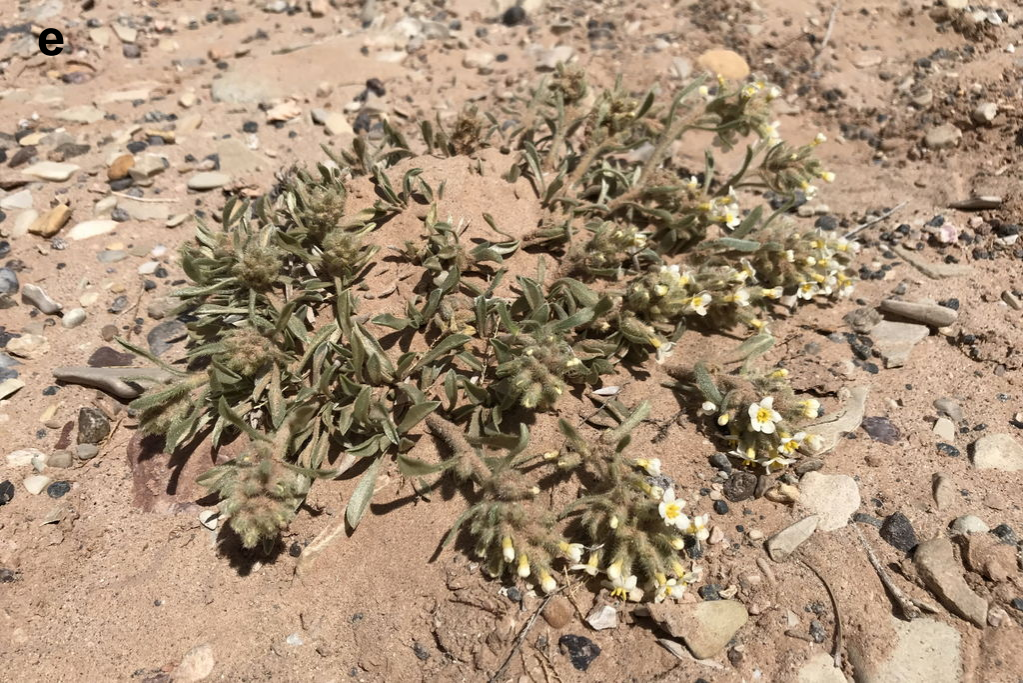
*Oreocarya flavoculata*, habit (*Sokoloff et al. 1268*).

**Figure 7f.**
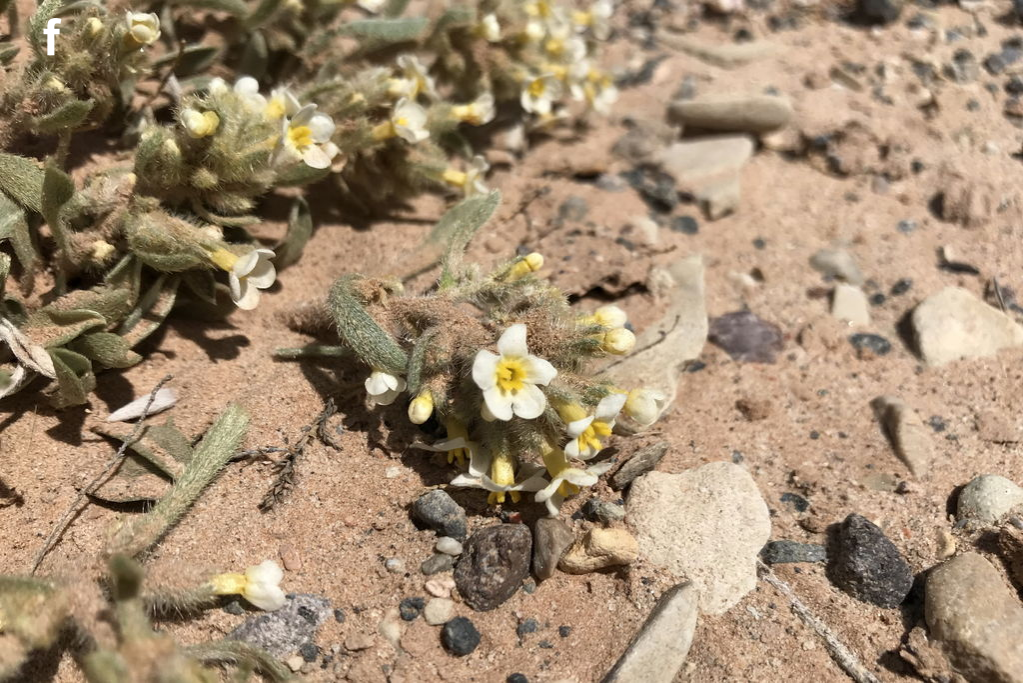
*Oreocarya flavoculata*, inflorescence (*Sokoloff et a. 1268*).

**Figure 8a.**
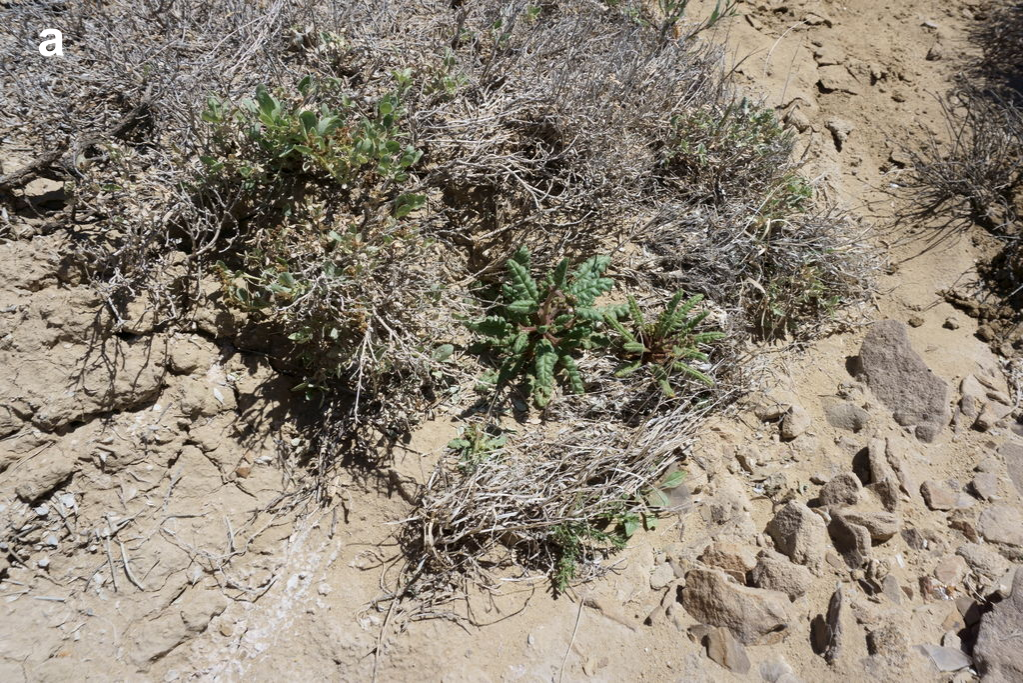
Phacelia crenulata var. corrugata, habitat (*Sokoloff et al. 1260*).

**Figure 8b.**
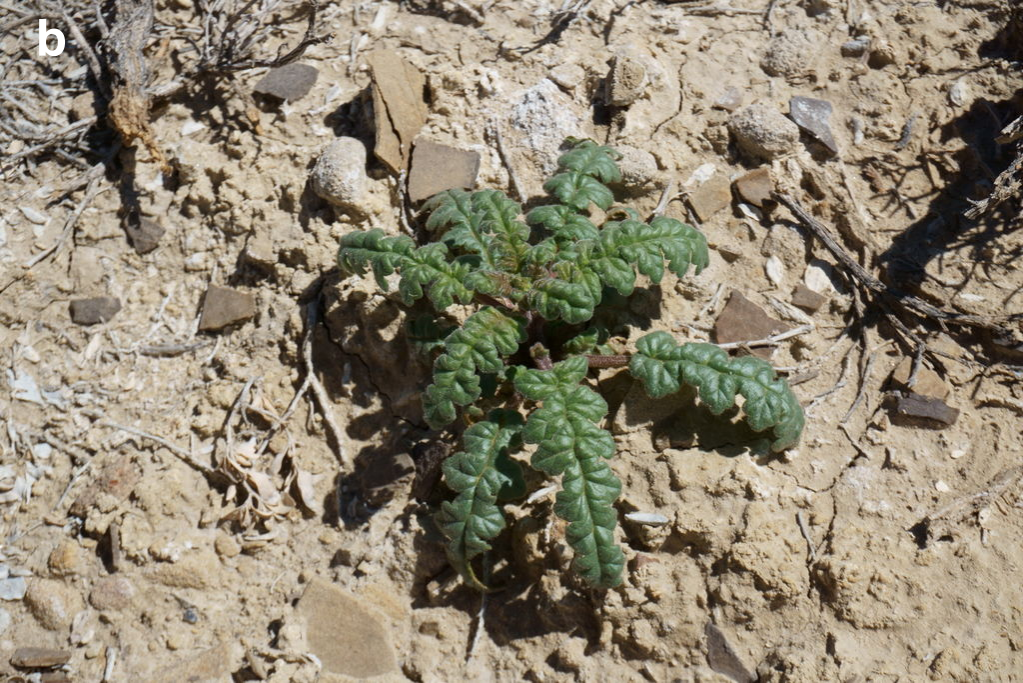
Phacelia crenulata var. corrugata, habit (*Sokoloff et al. 1260*).

**Figure 8c.**
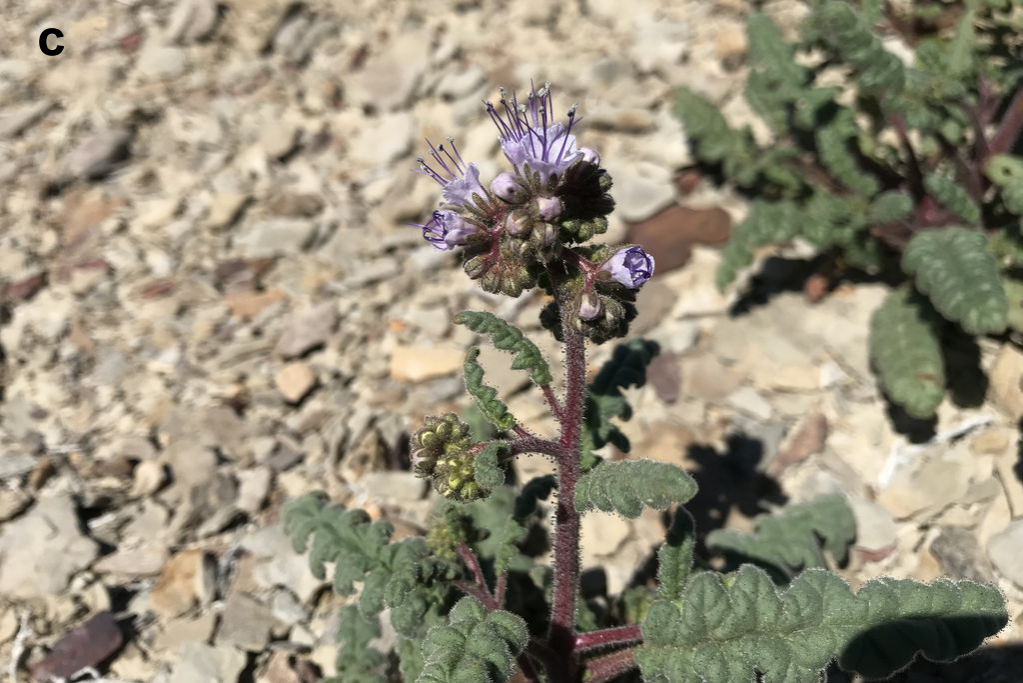
Phacelia crenulata var. corrugata, inflorescence (*Sokoloff et al. 1309*).

**Figure 8d.**
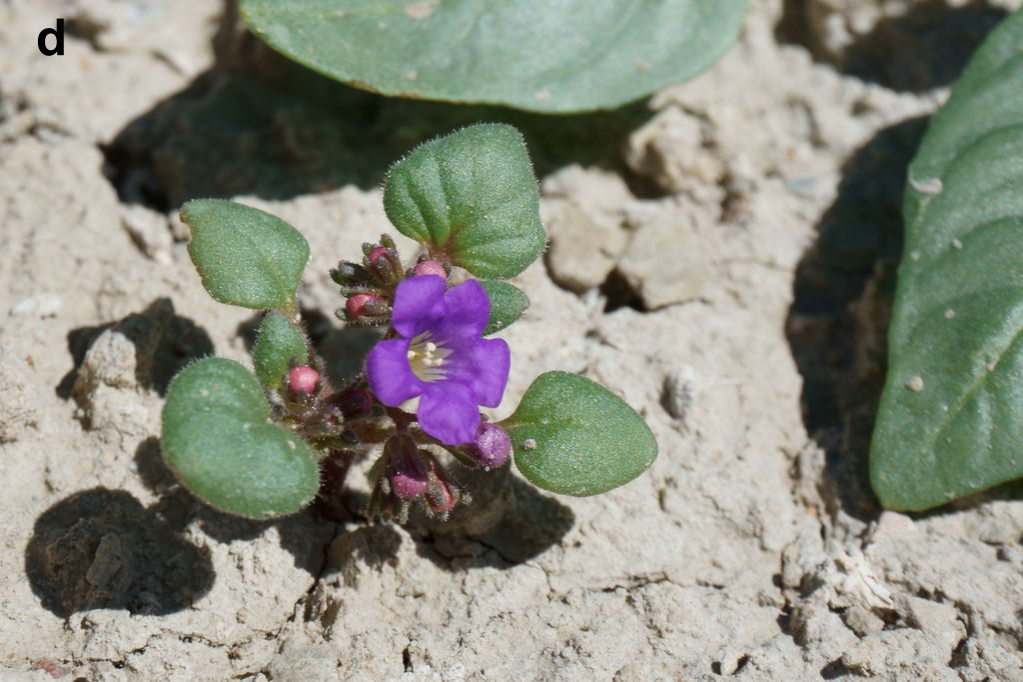
Phacelia demissa var. demissa, inflorescence (*Sokoloff et al. 1308*).

**Figure 8e.**
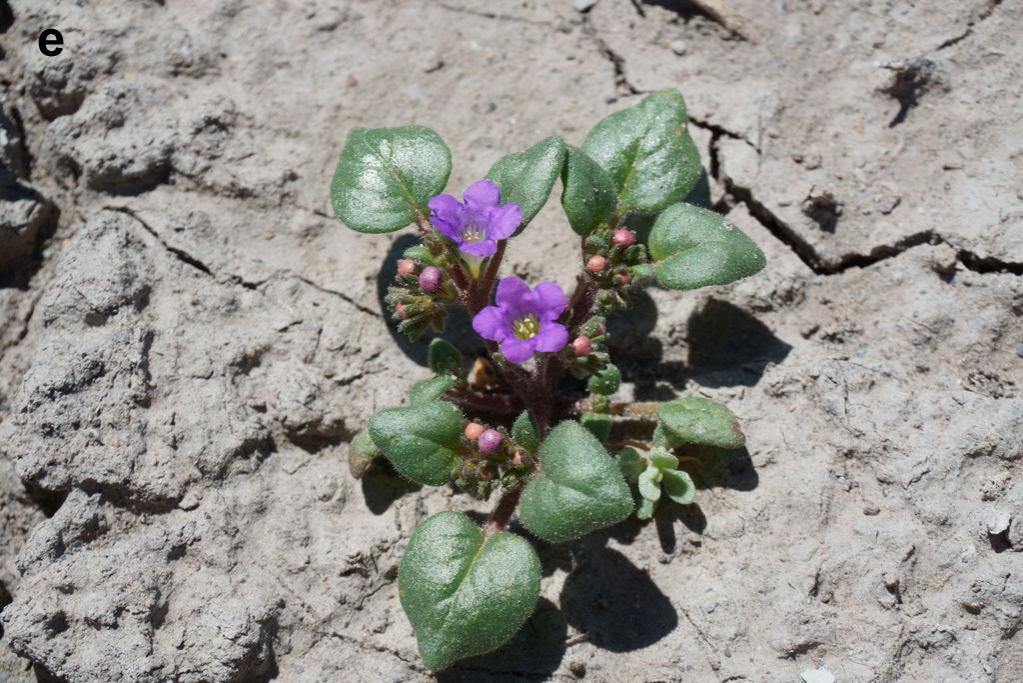
Phacelia demissa var. demissa, habit (*Sokoloff et al. 1312*).

**Figure 8f.**
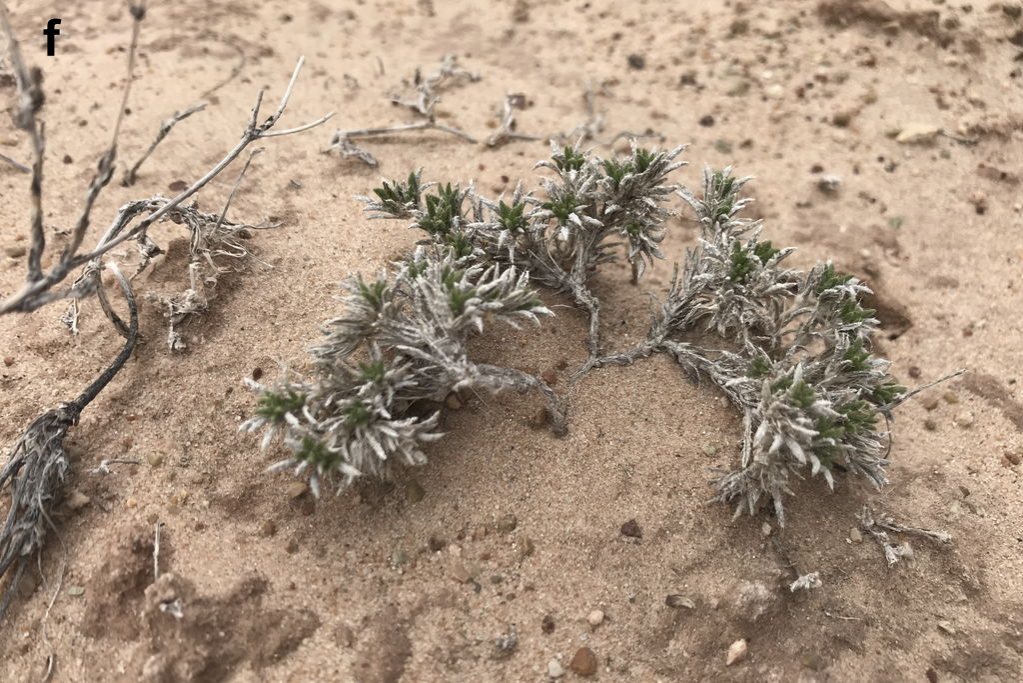
*Tiquilia latior*, habit (*Sokoloff et al. 1290*).

**Figure 9a.**
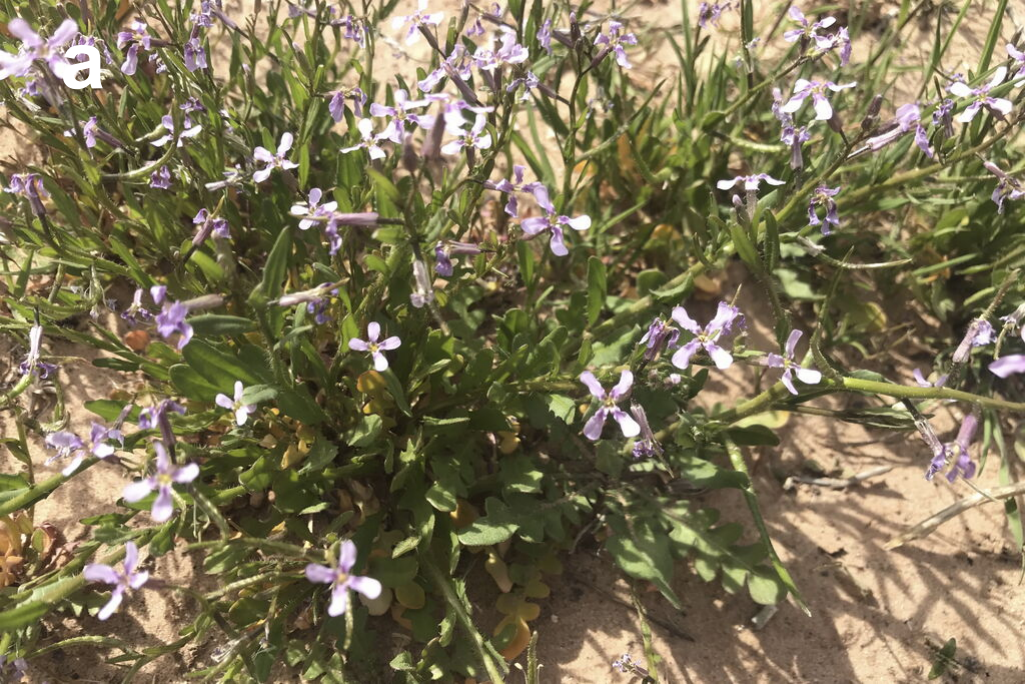
*Chorispora tenella*, habit (vicinity of Burpee Dinosaur Quarry).

**Figure 9b.**
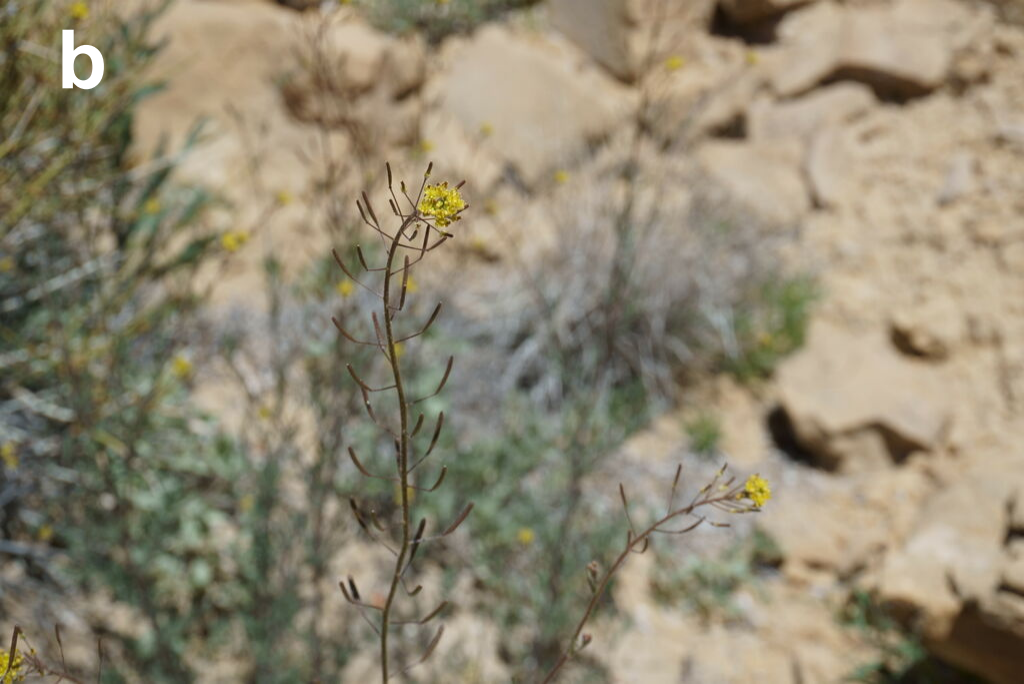
Descurainia pinnata subsp. brachycarpa, inflorescence (*Sokoloff et al. 1262*).

**Figure 9c.**
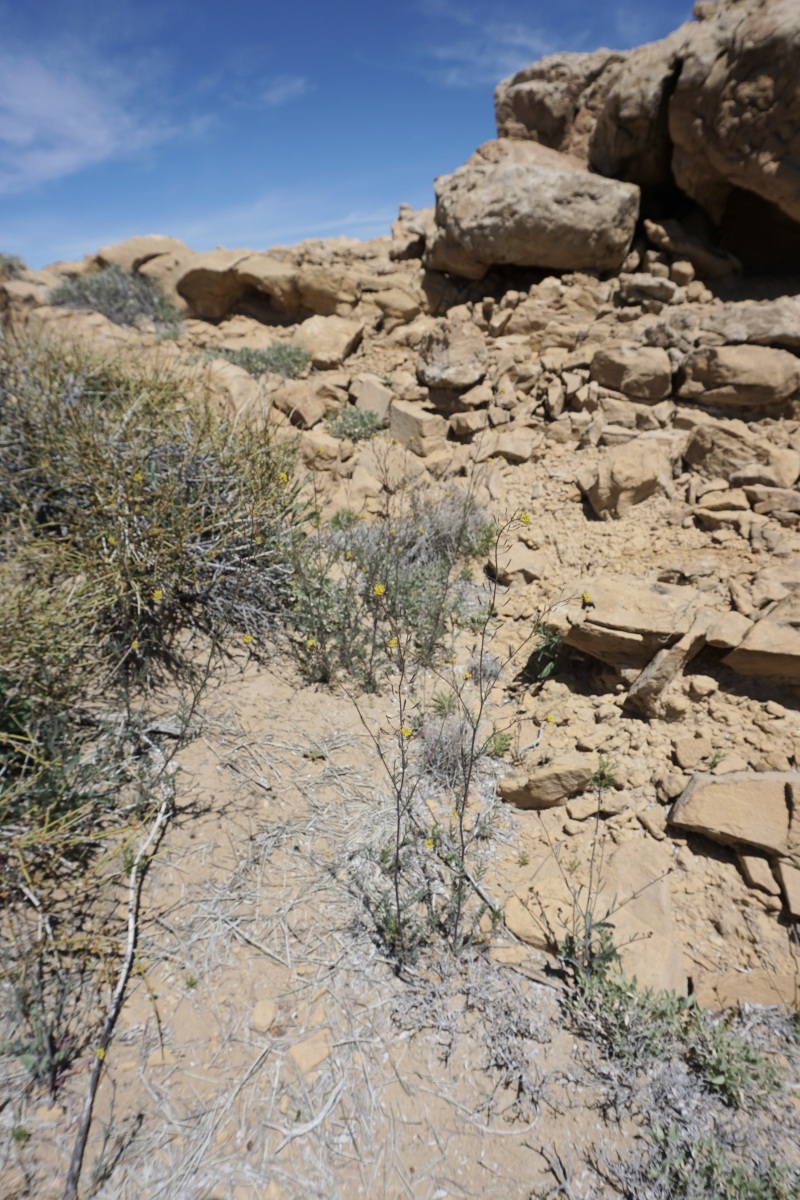
Descurainia pinnata subsp. brachycarpa, habit (*Sokoloff et al. 1262*).

**Figure 9d.**
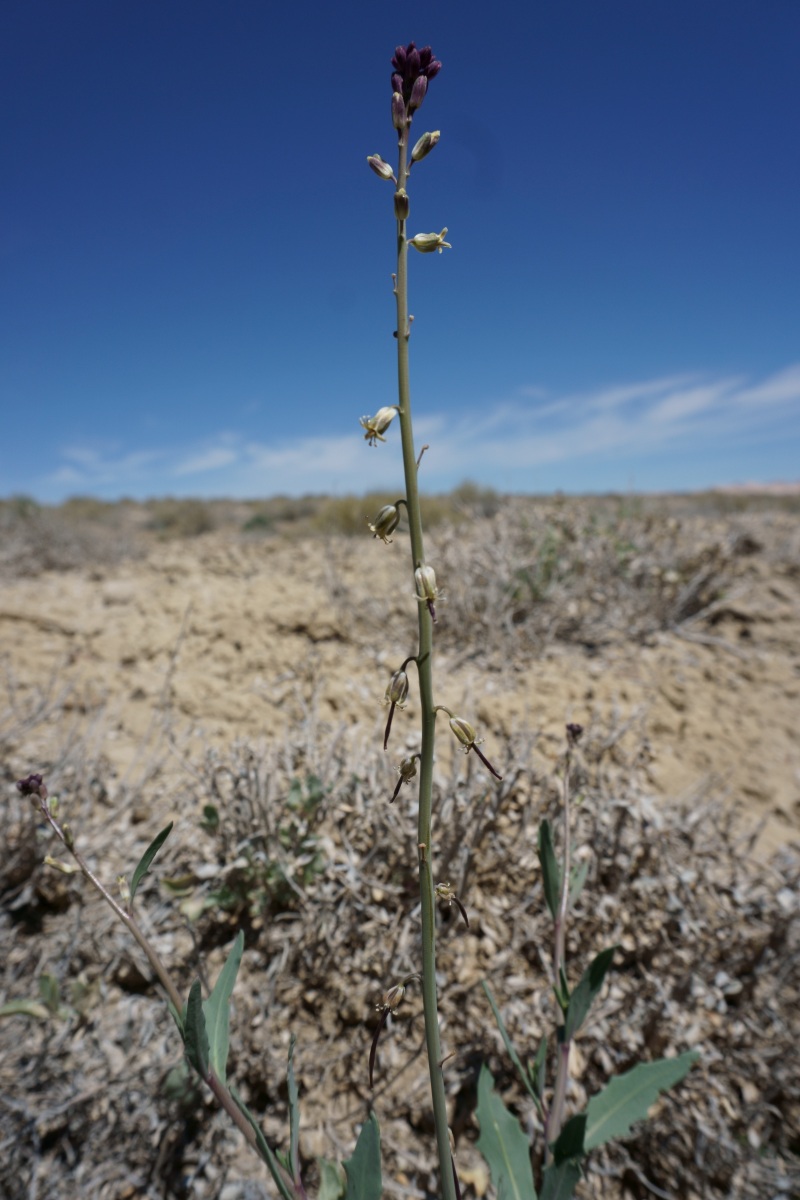
*Streptanthella longirostris*, inflorescence (*Sokoloff et al. 1261*).

**Figure 9e.**
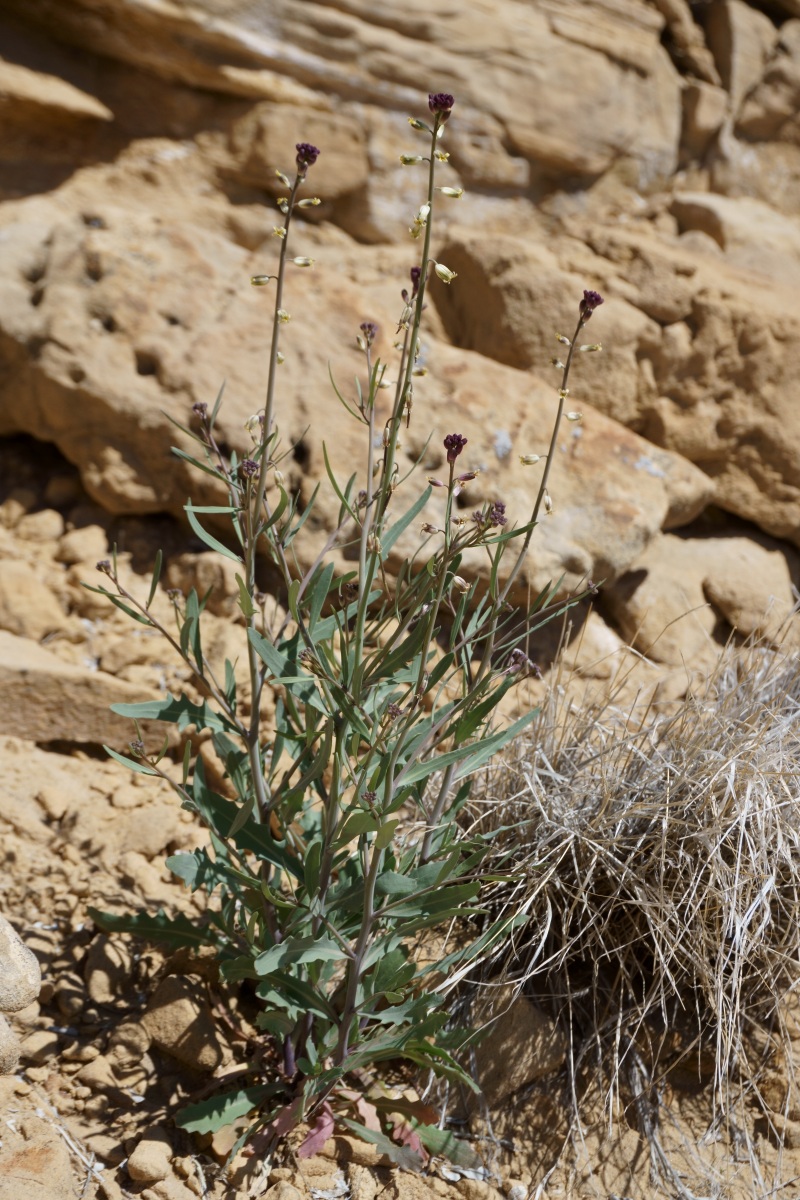
*Streptanthella longirostris*, habit (*Sokoloff et al. 1261*).

**Figure 9f.**
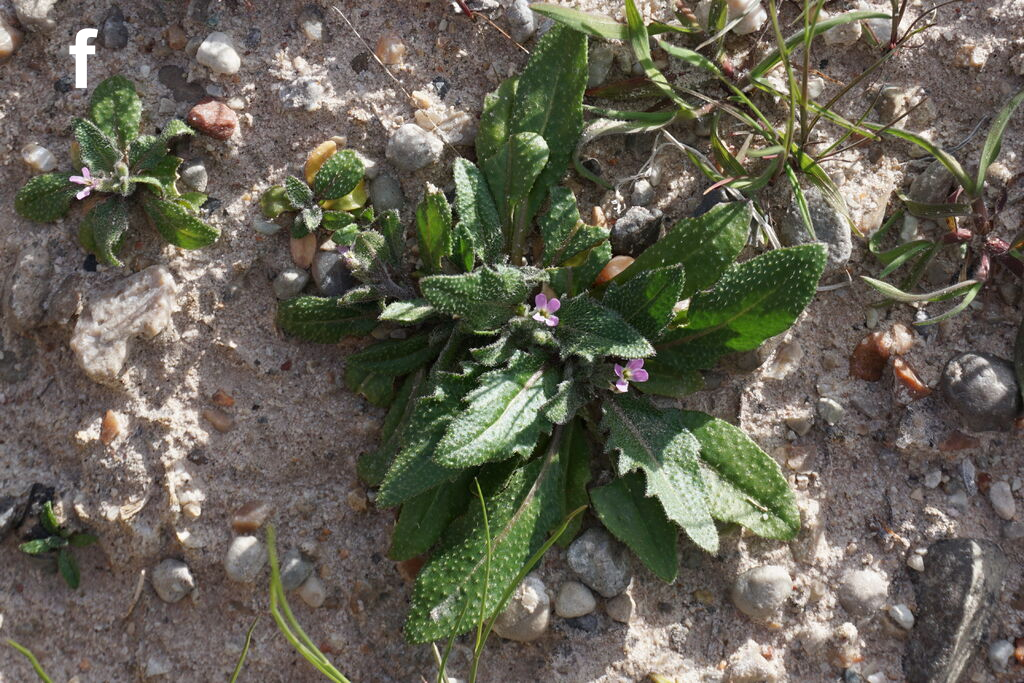
*Strigosella africana*, habit (*Sokoloff et al. 1278*).

**Figure 10a.**
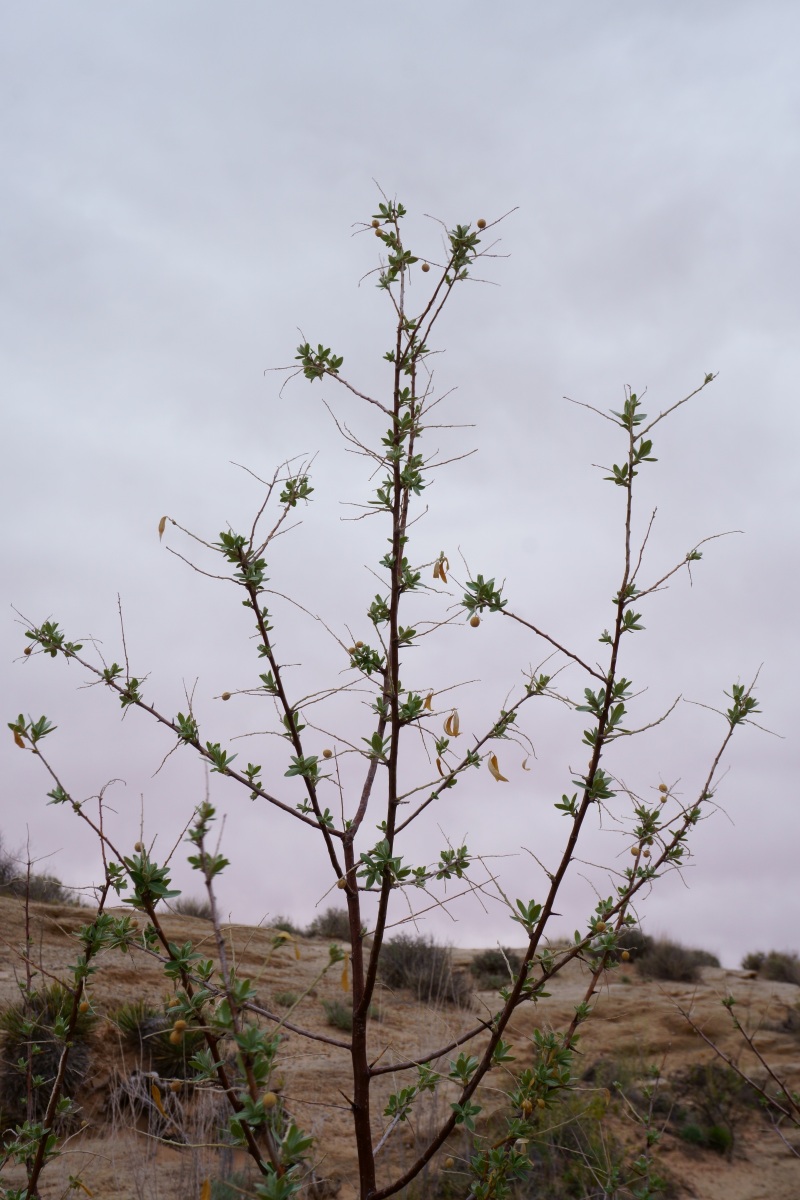
*Symphoricarpos longiflorus*, habit (*Sokoloff et al. 1296*).

**Figure 10b.**
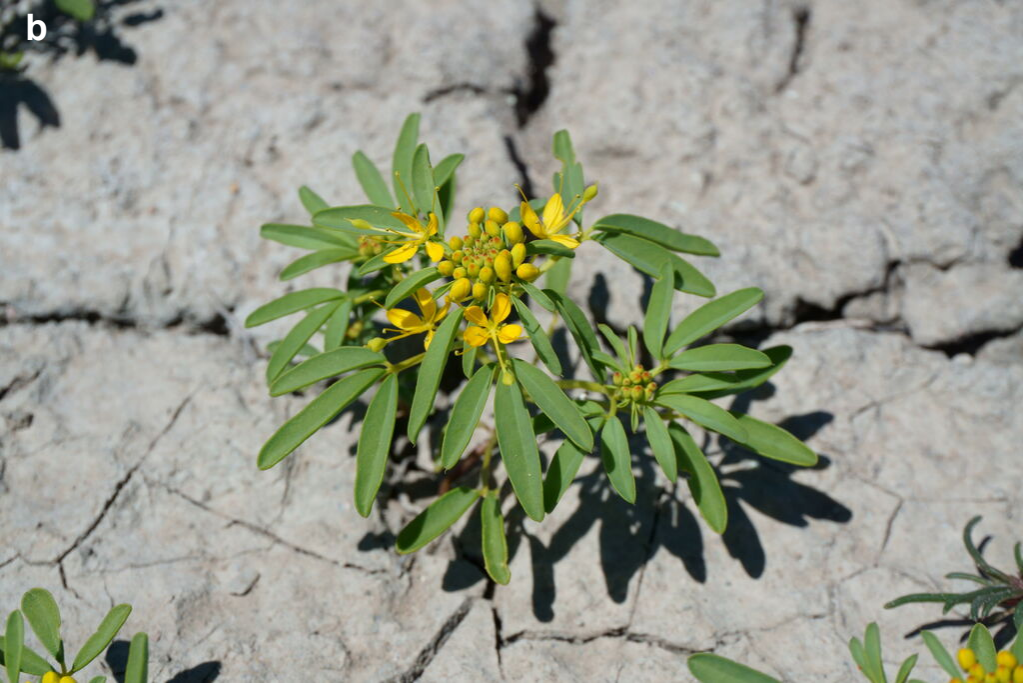
*Cleomella palmeriana*, habit (*Sokoloff et al. 1311*).

**Figure 11a.**
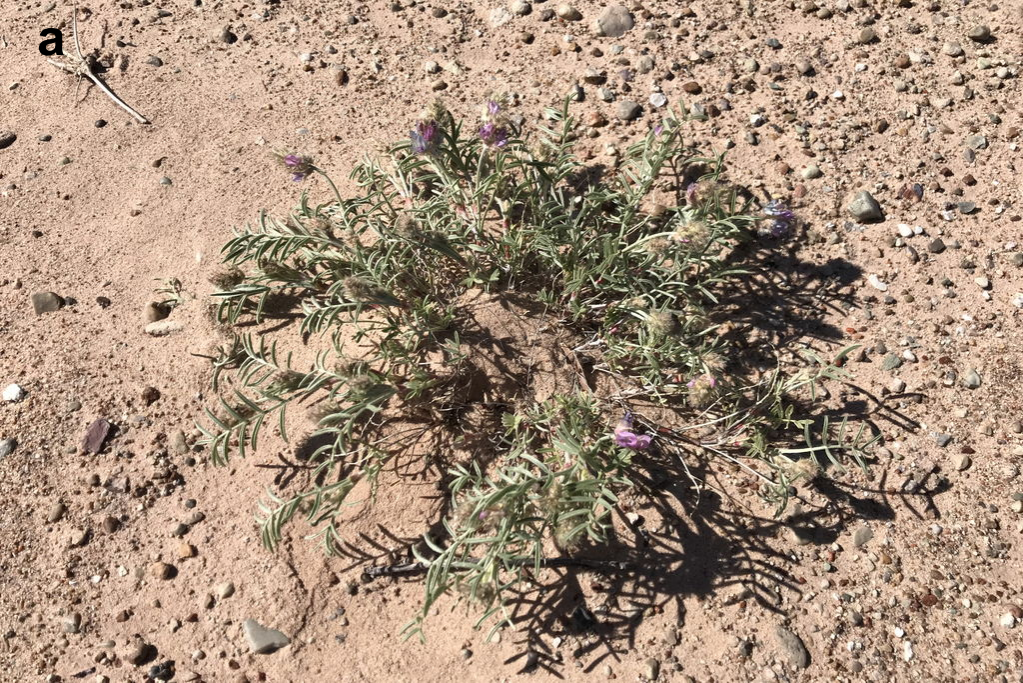
*Astragalus desperatus*, habit (*Sokoloff et al. 1317b*).

**Figure 11b.**
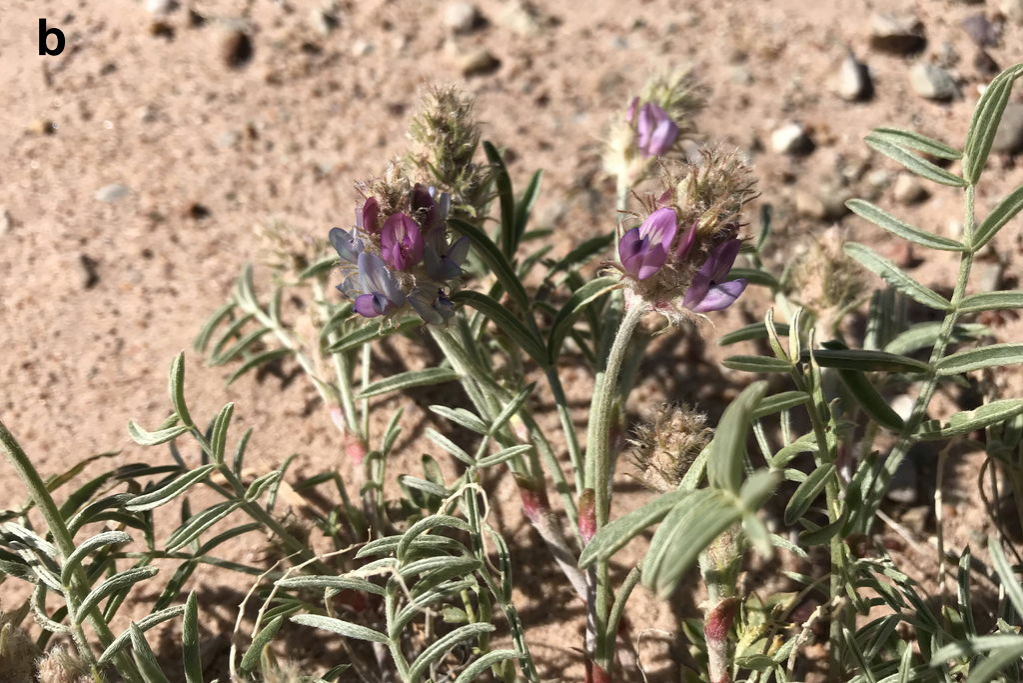
*Astragalus desperatus*, inflorescence (*Sokoloff et al. 1317b*).

**Figure 11c.**
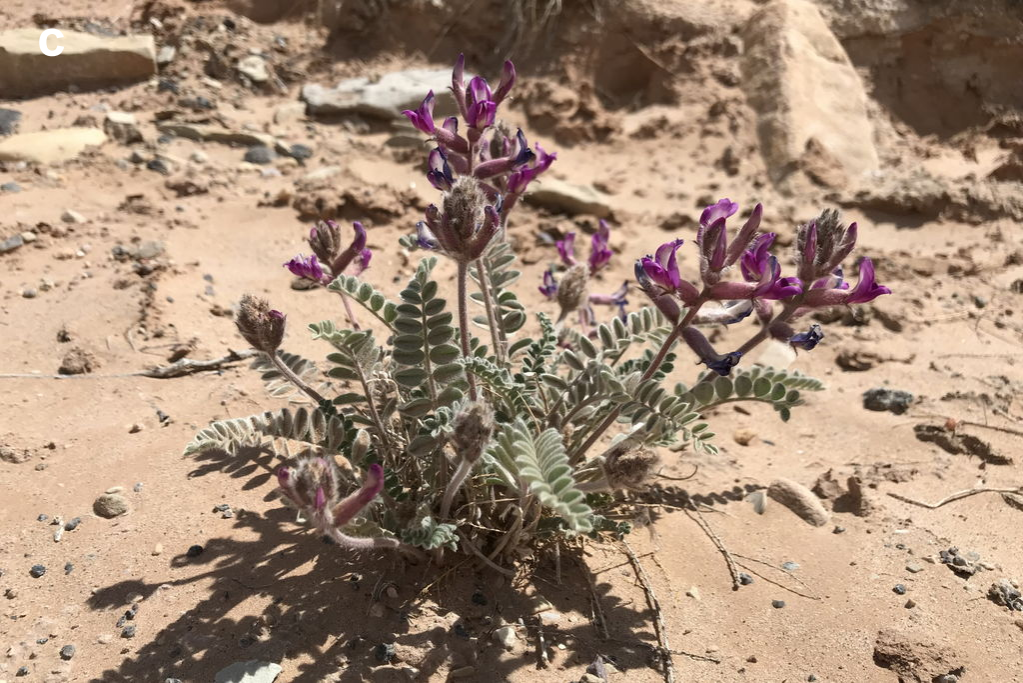
Astragalus mollissimus var. thompsoniae, habit (*Sokoloff et al. 1265*).

**Figure 11d.**
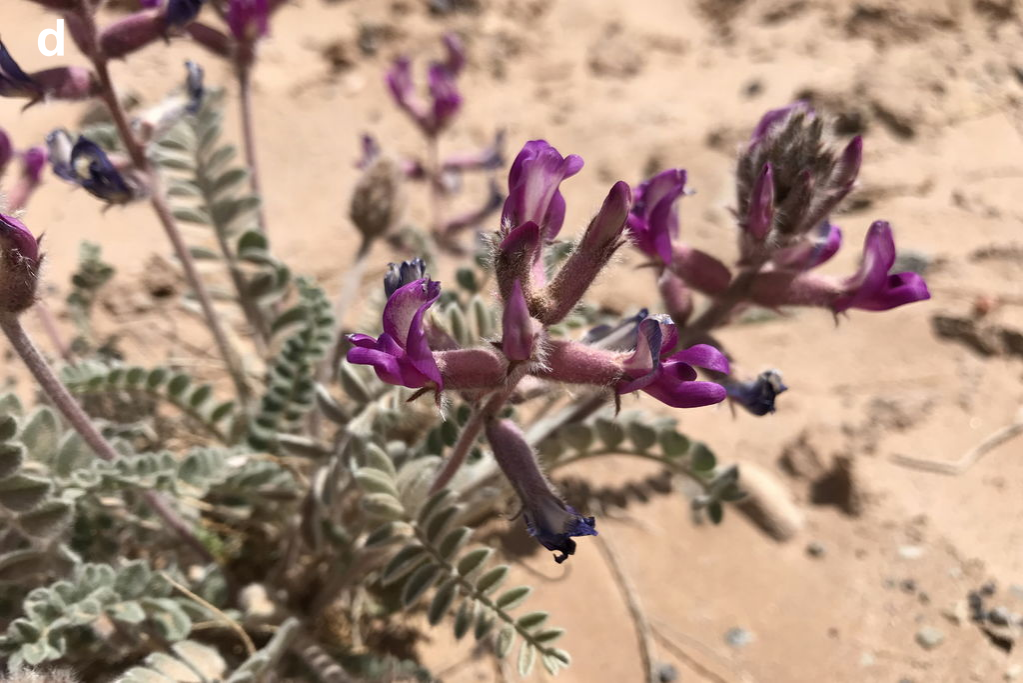
Astragalus mollissimus var. thompsoniae, inflorescence (*Sokoloff et al. 1265*).

**Figure 11e.**
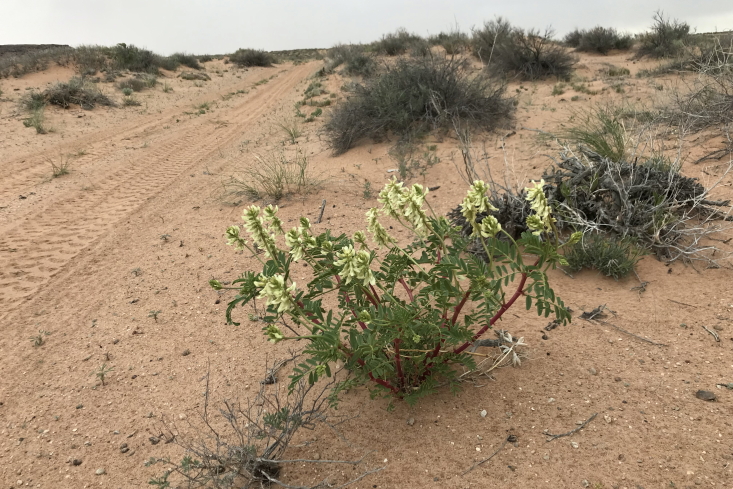
*Astragalus praelongus*, habit (*Sokoloff et al. 1301*).

**Figure 11f.**
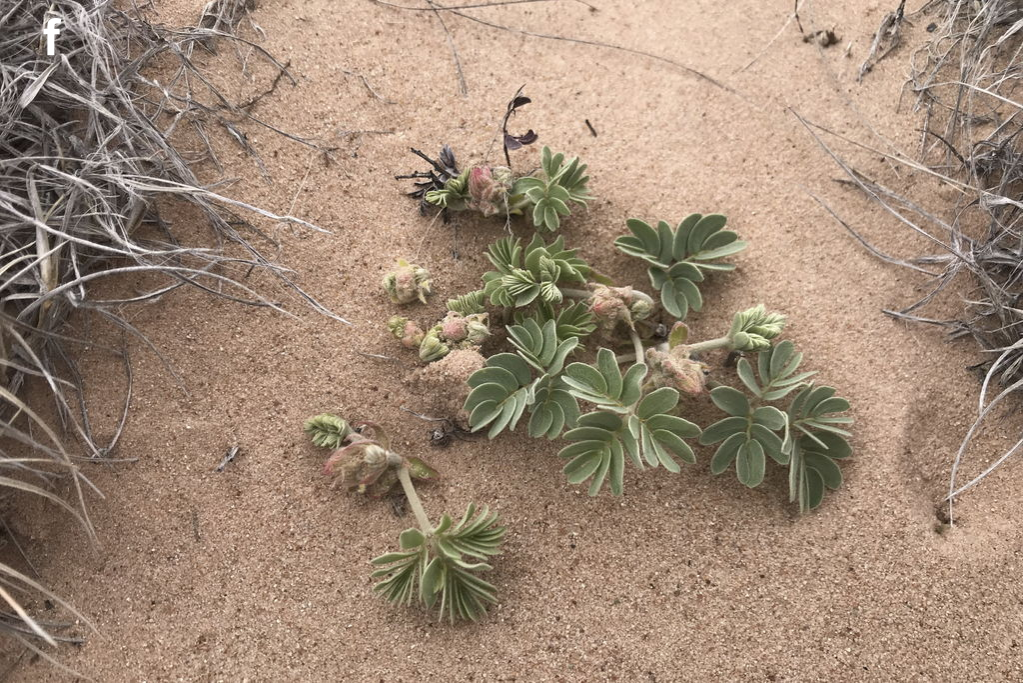
.*Hoffmannseggia repens*, habit (*Sokoloff et al. 1285*)

**Figure 12a.**
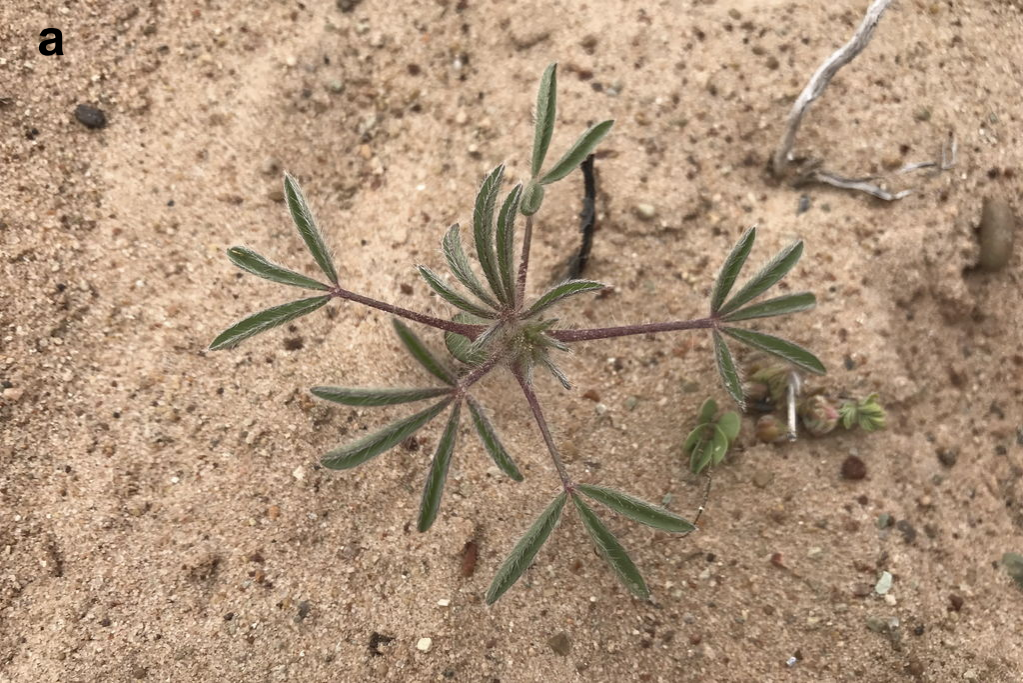
*Lupinus pusillus*, habit (*Sokoloff et al. 1287*).

**Figure 12b.**
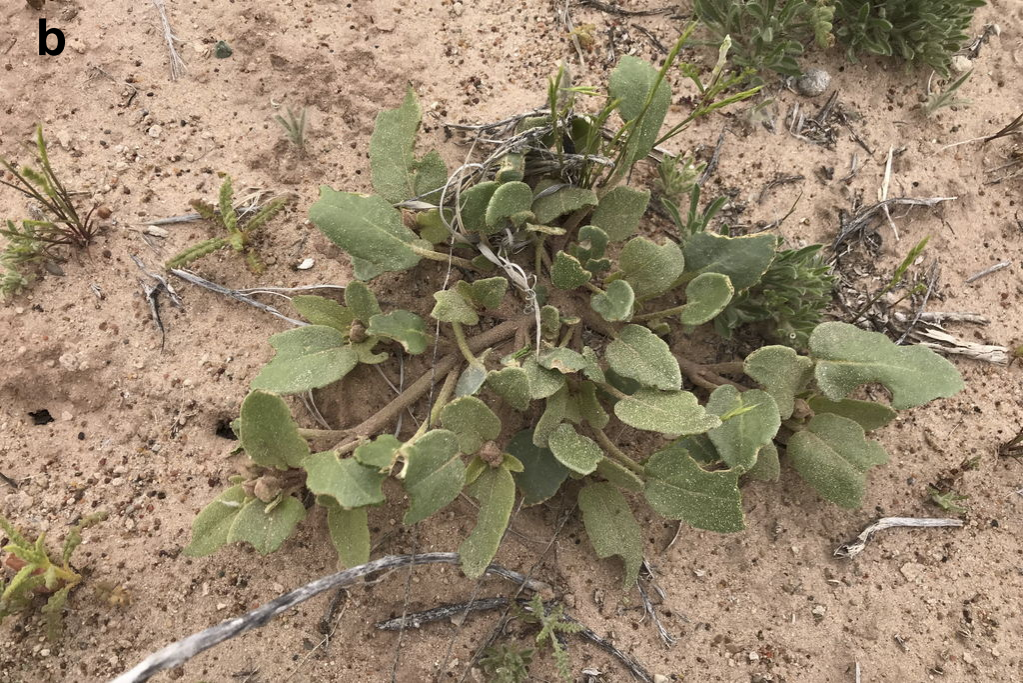
*Abronia elliptica*, habit (*Sokoloff et al. 1292*).

**Figure 12c.**
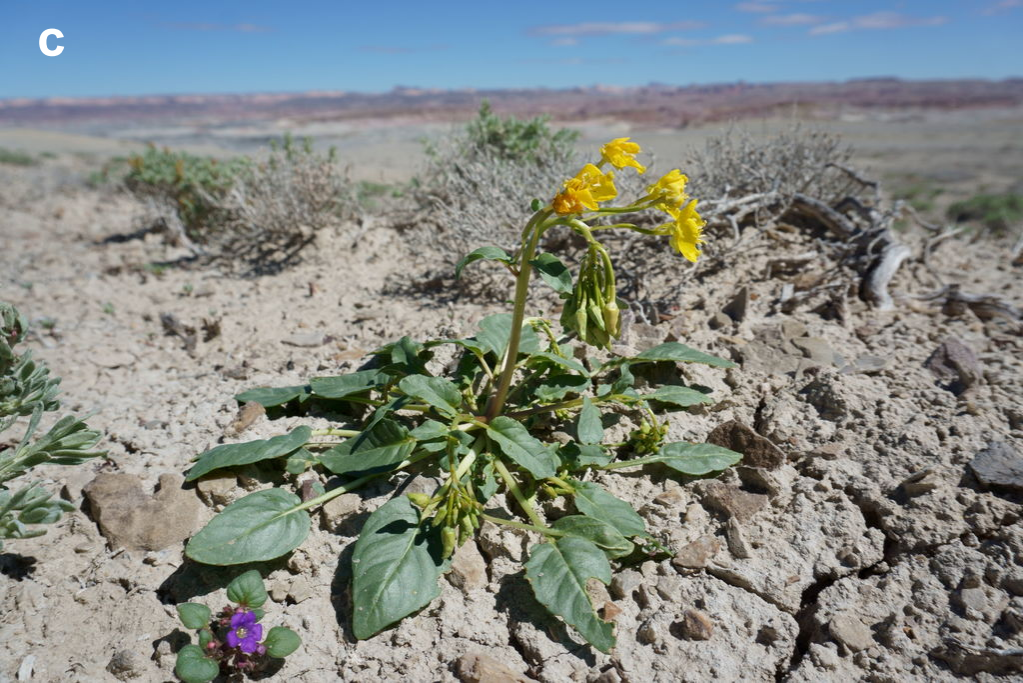
*Camissonia eastwoodiae*, habit (*Sokoloff et al. 1307*).

**Figure 12d.**
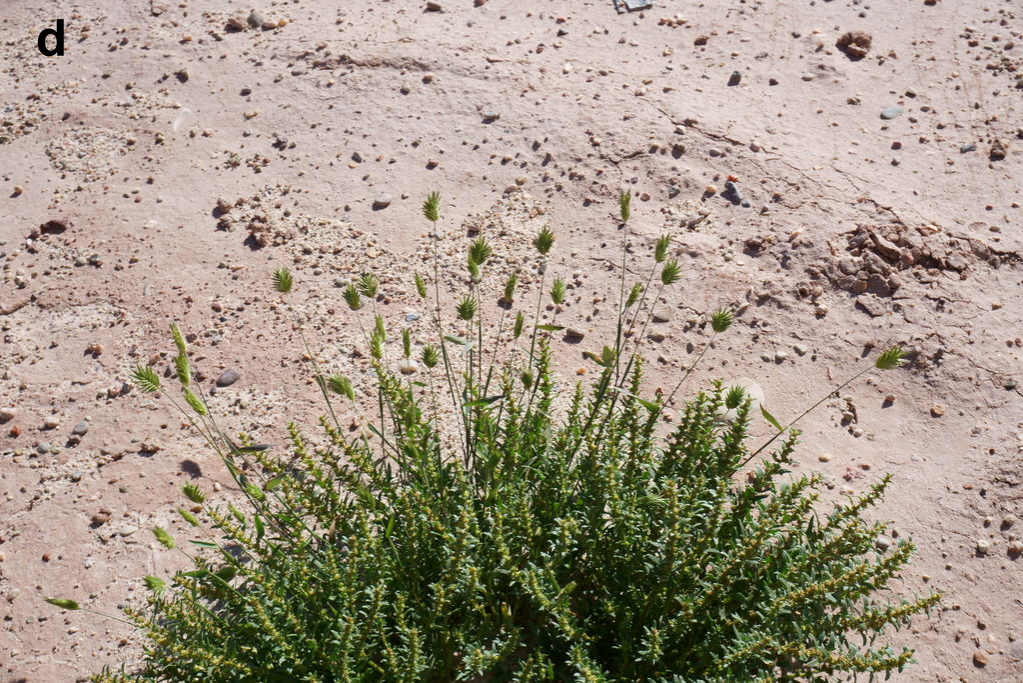
*Eremopyrum triticeum*, habit (*Sokoloff et al. 1321*).

**Figure 12e.**
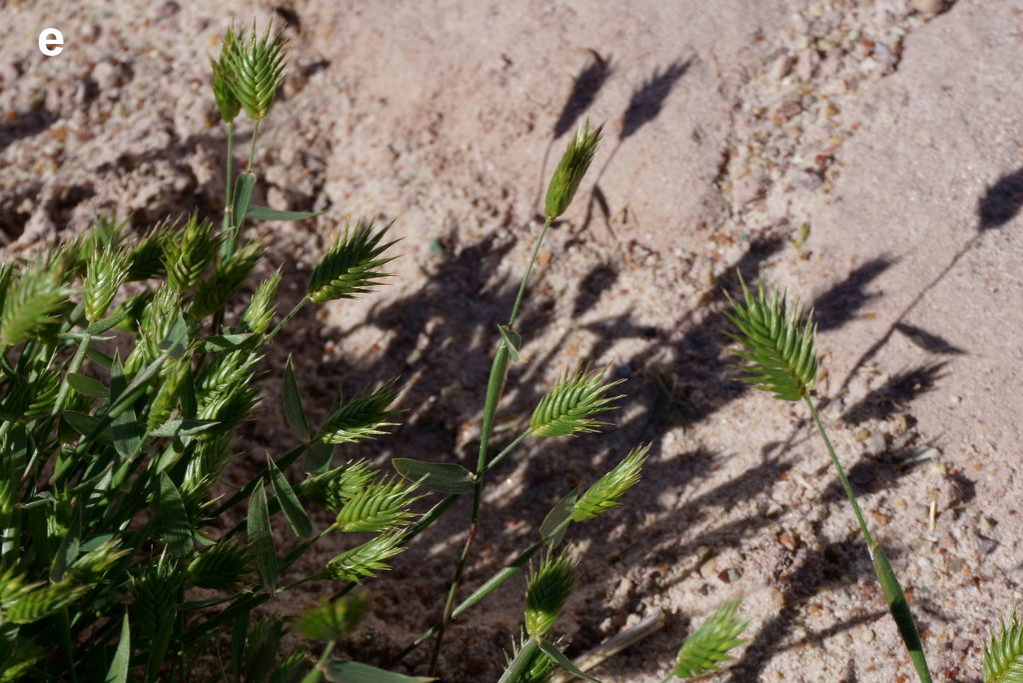
*Eremopyrum triticeum*, inflorescences (*Sokoloff et al. 1321*).

**Figure 12f.**
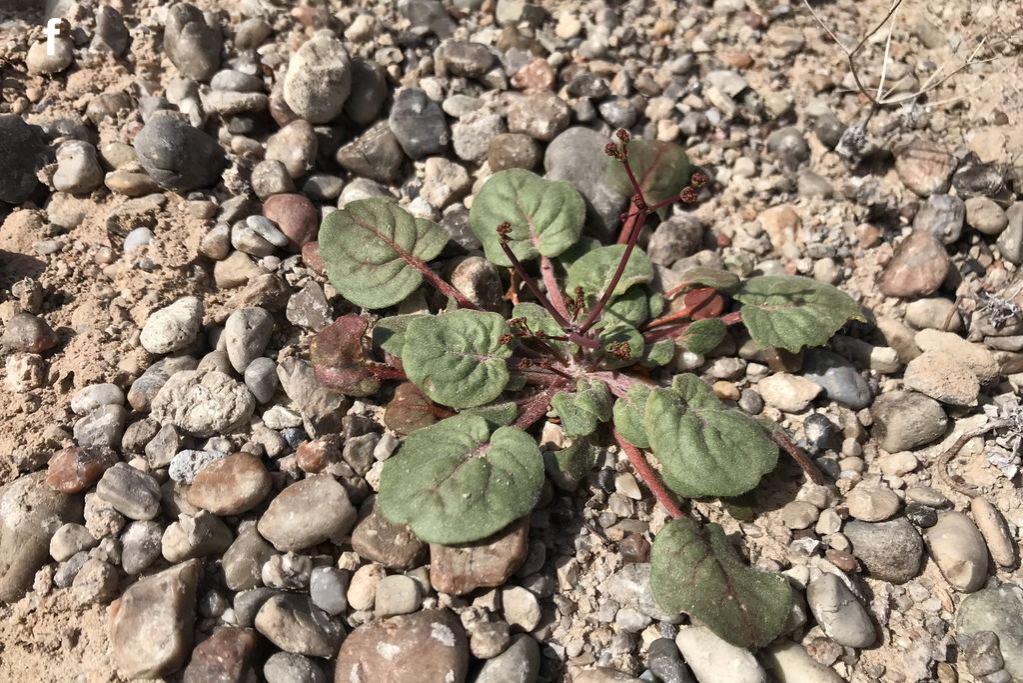
*Eriogonum wetherillii*, habit (*Sokoloff et al. 1272*).

**Table 1.** Collection Sites for Crew 210

**Collection Numbers**	**Date**	**Locality**	**Coordinates**	**Habitat**
1260-1264	14 April 2019	Emery County, "Valley of the Stars", 16 km from Highway 24 along Factory Bench Road.	38°30'6.48"N, 110°55'37.44"W	Sandstone bluffs with *Atriplex gardneri*, *Dasyochloa pulchella*, *Ephedra viridis*, *Artemisia tridentata*.
1265-1270	14 April 2019	Wayne County, Salt Wash at the end of Factory Bench Road, 20 km from Highway 24.	38°28'51.3"N, 110°57'58.62"W	Sandy shoreline of Salt Creek, dominated by *Tamarix ramosissima* and *Ericameria nauseosa*.
1271-1279	14 April 2019	Wayne County, area just south of Burpee Dinosaur Quarry at the end of Cow Dung Road, 5 km N of the Mars Desert Research Station.	38°27'5.42"N, 110°47'30.74"W	Sandy plains dominated by *Allium textile*, *Gutierrezia sarothrae*, *Ephedra viridis*.
1280-1291	16 April 2019	Wayne County, west side of Bureau of Land Management Road 1104, 2 km NE of the Mars Desert Research Station.	38°24'58.28"N, 110°46'17.4"W	Sandy plains dominated by *Artemisia filifolia* and *Scabrethia scabra*.
1292-1295, 1323	16 April 2019	Wayne County, south side of Bureau of Land Management Road 1104, 3.5 km NE of the Mars Desert Research Station.	38°25'14.84"N, 110°45'22.07"W	Rocky desert plain with basalt ejecta, with *Atriplex* sp. and *Sporobolus* sp.
1296-1300	16 April 2019	Wayne County, sandy wash south of Bureau of Land Management Road 1104, 3.75 km NE of the Mars Desert Research Station.	38°25'5.5"N, 110°45'5.5"W	Sheltered, sandy wash in rocky valley, with *Ericameria nauseosa* and *Artemisia tridentata*.
1301	16 April 2019	Wayne County, south side of Bureau of Land Management Road 1104, 3.5 km NE of the Mars Desert Research Station.	38°25'0.2"N, 110°45'54.4"W	Rocky desert plain with basalt ejecta, with *Atriplex* sp. and *Sporobolus* sp.
1302-1306	17 April 2019	Wayne County, east side of Cow Dung Road, on a ridge just east of turnoff onto Bureau of Land Management Road 1572, 4 km N of the Mars Desert Research Station	38°26'30.2"N, 110°47'37.3"W	Rocky ridge with *Atriplex* sp.
1307-1309	17 April 2019	Wayne County, crossroads of Bureau of Land Management Road 1572 and 1575, 4 km NW of the Mars Desert Research Station.	38°26'21.2"N, 110°48'57.6"W	Clay Mancos Shale ridge with *Atriplex corrugata*.
1310-1314	17 April 2019	Wayne County, "Copernicus Valley" along Bureau of Land Management Highway 0157, 5.7 km NW of the Mars Desert Research Station.	38°27'26.9"N, 110°48'5.5"W	Silty clay flats with *Sarcobatus vermiculatus*.
1315-1317	17 April 2019	Wayne County, "Hab Ridge", sandstone ridge at edge of Lower Blue Hills, immediately west of the Mars Desert Research Station.	38°24'40.9"N, 110°47'42.4"W	Dakota Sandstone/Mancos Shale substrate with *Aristida purpurea* and *Gutierrezia sarothrae*.
1317b-1319	17 April 2019	Wayne County, North Pinto Hills, desert flats immediately east of Cow Dung Road, 1.75 km N of Highway 24.	38°23'13.97"N, 110°46'13.51"W	Flat sandy plains dominated by *Allium textile* and *Sarcobatus vermiculatus*.
1320-1322	17 April 2019	Wayne County, area immediately surrounding the Mars Desert Research Station.	38°24'22.71"N, 110°47'30.79"W	Red clay flats.

**Table 2.** New plant taxa documented by Crew 210 for the Mars Desert Research Station area, arranged according to the APG IV linear sequence. Taxa with an asterisk* were photo-documented only.

monocots	Asparagales	Amaryllidaceae	*Allium macropetalum* Rydb
		Asparagaceae	*Eremocrinum albomarginatum* (M.E.Jones) M.E.Jones
			*Yucca harrimaniae* Trel.
	Poales	Poaceae	*Eremopyrum triticeum* (Gaertn.) Nevski
			Vulpia octoflora (Walter) Rydb. var. octoflora
eudicots	Fabales	Fabaceae	Astragalus mollissimus var. thompsoniae (S. Watson) Barneby
			*Astragalus pardalinus* (Rydb.) Barneby
			*Astragalus praelongus* E. Sheld.
			*Astragalus woodruffii* M.E. Jones
			*Hoffmannseggia repens* (Eastw.) Cockerell
			*Lupinus pusillus* Pursh
	Malpighiales	Salicaceae	Populus fremontii S. Watson subsp. fremontii
	Myrtales	Onagraceae	*Camissonia eastwoodiae* (Munz) P.H. Raven
			*Oenothera pallida* Lindl.
	Sapindales	Anacardiaceae	Rhus trilobata Nutt. var. trilobata
	Brassicales	Cleomaceae	*Cleomella palmeriana* M.E. Jones
		Brassicaceae	*Chorispora tenella* (Pall.) DC.*
			Descurainia pinnata subsp. brachycarpa (Richardson) Detling
			Stanleya pinnata (Pursh) Britton var. pinnata
			*Streptanthella longirostris* (S.Watson) Rydb.
			*Strigosella africana* (L.) Botsch.
	Caryophyllales	Polygonaceae	*Eriogonum gordonii* Benth.
			*Eriogonum wetherillii* Eastw.
		Amaranthaceae	*Atriplex argentea* Nutt.
			Atriplex canescens (Pursh) Nutt. var. canescens
			*Blitum nuttallianum* Schult.
		Nyctaginaceae	*Abronia elliptica* A. Nelson
		Cactaceae	*Pediocactus* Britton & Rose*
			*Sclerocactus* Britton & Rose*
	Cornales	Loasaceae	*Mentzelia pterosperma* Eastw.
	Boraginales	Boraginaceae	Cryptantha crassisepala var. elachantha I.M. Johnst.
			*Oreocarya flava* A.Nelson
			*Oreocarya flavoculata* A. Nelson
			*Phacelia corrugata* A. Nelson
			Phacelia demissa A. Gray var. demissa
			*Tiquilia latior* (I.M. Johnston) A. Richards.
	Asterales	Asteraceae	*Chaenactis stevioides* Hook. & Arn.
			*Malacothrix sonchoides* (Nutt.) Torr. & A. Gray
			*Prenanthella exigua* (A. Gray) Rydb.
			*Tetradymia glabrata* Torr. & A. Gray
	Dipsacales	Caprifoliaceae	*Symphoricarpos longiflorus* Gray.
	Apiales	Apiaceae	*Cymopterus glomeratus* (Nutt.) Raf.

## References

[B5924692] Al-Shehbaz I. A., Flora of North America Editorial Committee eds. 1993+ (2010). *
Stanleya
*. Flora of North America North of Mexico.

[B5924705] Al-Shehbaz I. A., Flora of North America Editorial Committee eds. 1993+ (2010). *
Strigosella
*. Flora of North America North of Mexico.

[B5924718] Al-Shehbaz I. A., Flora of North America Editorial Committee eds. 1993+ (2010). *
Streptanthella
*. Flora of North America North of Mexico.

[B5924731] Al-Shehbaz I. A., Flora of North America Editorial Committee eds. 1993+ (2010). *
Chorispora
*. Flora of North America North of Mexico.

[B5532284] Al-Shehbaz I. A., German D. A., Mummenhoff K., Moazzeni H. (2014). Systematics, tribal placements, and synopses of the *Malcolmia* s.l. segregates (Brassicaceae). Harvard University Botany Libraries.

[B5349473] Andersen B. A. (1996). Desert Plants of Utah.

[B5533832] Banner R., Pratt M., Bowns J. (2011). Grasses and grasslike plants of Utah, a field guide.

[B5533808] Barkworth M. E., Dewey D. R., Atkins R. J. (1983). New generic concepts in the Triticeae of the Intermountain Region: key and comments. The Great Basin Naturalist.

[B5349482] Barneby R. C. (1964). Atlas of North American *Astragalus*.

[B5532294] Botschantzev V. P. (1972). The genus *Strigosella* Boiss. and its relation to the genus *Malcolmia* R. Br. (Cruciferae). Botanicheskiy Zhurnal (Moscow & Leningrad).

[B5924593] Chambers K. L., Flora of North America Editorial Committee eds. 1993+ (2006). *
Prenanthella
*. Flora of North America North of Mexico.

[B5924624] David W. S., Flora of North America Editorial Committee eds. 1993+ (2006). *
Malacothrix
*. Flora of North America North of Mexico.

[B5700428] Direito Susana O. L., Ehrenfreund Pascale, Marees Andries, Staats Martijn, Foing Bernard, Röling Wilfred F. M. (2011). A wide variety of putative extremophiles and large beta-diversity at the Mars Desert Research Station (Utah). International Journal of Astrobiology.

[B5924744] Eckenwalder J. E., Flora of North America Editorial Committee eds. 1993+ (2010). *
Populus
*. Flora of North America North of Mexico.

[B5535001] Evans Margaret E. K., Hearn David J., Hahn William J., Spangle Jennifer M., Venable D. Lawrence (2005). Climate and life-history evolution in Evening Primroses (*Oenothera*, Onagraceae): a phylogenetic comparative analysis. Evolution.

[B5661305] Feist L. J., Parker D. R. (2001). Ecotypic variation in selenium accumulation among populations of *Stanleya
pinnata*. New Phytologist.

[B5353386] Fertig W (2009). Annotated checklist of vascular flora: Capitol Reef National Park. Natural Resource Technical Report NPS/NCPN/NRTR–2009/154..

[B5670852] Flora of North America Editorial Committee eds. (1993). Flora of North America North of Mexico.

[B5700460] Foing B. H., Stoker C., Ehrenfreund P. (2011). Astrobiology field research in Moon/Mars analogue environments. International Journal of Astrobiology.

[B5924666] Frederiksen S., Flora of North America Editorial Committee eds. 1993+ (2007). *
Eremopyrum
*. Flora of North America North of Mexico.

[B5297882] Fuentes-Bazan Susy, Uotila Pertti, Borsch Thomas (2012). A novel phylogeny-based generic classification for *Chenopodium* sensu lato, and a tribal rearrangement of Chenopodioideae (Chenopodiaceae). Willdenowia - Annals of the Botanic Garden and Botanical Museum Berlin-Dahlem.

[B5535036] Galloway L. A. (1975). Systematics of the North American Desert Species of *Abronia* and *Tripterocalyx* (Nyctaginaceae). Brittonia.

[B5923876] Galloway L. A., Flora of North America Editorial Committee eds. 1993+ (2003). *
Abronia
*. Flora of North America North of Mexico.

[B5923820] Goodson B. E., Al-Shehbaz I. A., Flora of North America Editorial Committee eds. 1993+ (2010). *
Descurainia
*. Flora of North America North of Mexico.

[B5297892] Harris J. G. (1983). A vascular flora of the San Rafael Swell, Utah.. Great Basin Naturalist.

[B5412517] Hasenstab-Lehman K. E., Simpson M. G. (2012). Cat's Eyes and Popcorn Flowers: Phylogenetic systematics of the genus *Cryptantha* s. l. (Boraginaceae). Systematic Botany.

[B5923848] Hess W. J., Robbins R. L., Flora of North America Editorial Committee eds. 1993+ (2002). *
Yucca
*. Flora of North America North of Mexico.

[B5412537] Higgins L. C. (1971). A revision of Cryptantha
subgenus
Oreocarya. Brigham Young University Science Bulletin, Biological Series.

[B5661187] Higgins L. C. (1979). Boraginaceae of the Southwestern United States. The Great Basin Naturalist.

[B5353395] Hill M, Ayers T (2009). Vascular plant inventory of Glen Canyon National Recreation Area. Natural Resource Technical Report NPS/SCPN/NRTR—2009/264.

[B5924105] Holmgren N. H., Flora of North America Editorial Committee eds. 1993+ (2003). *
Monolepis
*. Flora of North America North of Mexico.

[B5535684] Holmgren P. K. (2004). Lectotypifications and a new combination in western North American Cleomaceae. Brittonia.

[B5532304] Ivalú Cacho N., Millie Burrell A., Pepper A. E., Strauss S. Y. (2014). Novel nuclear markers inform the systematics and the evolution of serpentine use in *Streptanthus* and allies (Thelypodieae, Brassicaceae). Molecular Phylogenetics and Evolution.

[B5442513] Jennings P. W., Reeder S. K., Hurley J. C., Robbins J. E., Holian S. K., Holian A., Lee P., Pribanic J. A.S., Hull M. (1978). Toxic constituents and hepatotoxicity of the plant *Tetradymia
glabrata* (Asteraceae). Effects of Poisonous Plants on Livestock.

[B5412457] Johnston I. M. (1959). Some noteworthy American borages. Wrightia.

[B5442528] Kyhos D. W. (1965). The independent aneuploid origin of two species of *Chaenactis* (Compositae) from a common ancestor. Evolution.

[B5353453] Lee J., Baldwin B. G., Gottlieb L. D. (2002). Phylogeny of *Stephanomeria* and related genera (Compositae-Lactuceae) based on analysis of 18S-26S nuclear rDNA ITS and ETS sequences. American Journal of Botany.

[B5661335] Lichvar R. W. (1983). Evaluation of varieties in *Stanleya
pinnata* (Cruciferae). The Great Basin Naturalist.

[B5661294] Lonard R. I., Gould F. W. (1974). The North American species of *Vulpia* (Graminae). Madroño.

[B5924679] Lonard R. I., Flora of North America Editorial Committee eds. 1993+ (2007). *
Vulpia
*. Flora of North America North of Mexico.

[B5412507] Mabry M. E., Simpson M. G. (2018). Evaluating the monophyly and biogeography of *Cryptantha* (Boraginaceae). Systematic Botany.

[B5700470] Martins Z., Sephton M. A., Foing B. H., Ehrenfreund P. (2011). Extraction of amino acids from soils close to the Mars Desert Research Station (MDRS), Utah. International Journal of Astrobiology.

[B5923835] McNeal D. W. Jr, Jacobsen T. D., Flora of North America Editorial Committee eds. 1993+ (2002). *
Allium
*. Flora of North America North of Mexico.

[B5661197] Moore M. J., Jansen R. K. (2007). Origins and biogeography of gypsophily in the Chihuahuan Desert plant group Tiquilia
subg.
Eddya (Boraginaceae). Systematic Botany.

[B5924653] Morefield J. D., Flora of North America Editorial Committee eds. 1993+ (2006). *
Chaenactis
*. Flora of North America North of Mexico.

[B5532090] Neese E., Welsh S. L. (1985). New variety of *Yucca
harrimaniae* (Agavaceae) from Utah. Great Basin Naturalist.

[B5924783] Parfitt B. D., Gibson A. C., Flora of North America Editorial Committee eds. 1993+ (2003). Cactaceae. Flora of North America North of Mexico.

[B5412527] Payson Edwin Blake (1927). A Monograph of the section
Oreocarya of *Cryptantha*. Annals of the Missouri Botanical Garden.

[B5700989] Persaud R., Robles S. R., Clarke J. D., Dawson S., Mann G. A., Waldie J., Piechocinski S., Roesch J. (2003). Expedition One- A Mars Analog Research Station 30-day mission. American Astronautical Society Science and Technology Series.

[B5700440] Rai Balwant, Kaur Jasdeep (2012). Mental and physical workload, salivary stress biomarkers and taste perception: Mars Desert Research Station expedition. North American Journal of Medical Sciences.

[B5533864] Raven P. H. (1969). A revision of the genus *Camissonia* (Onagraceae). Contributions from the United States National Herbarium.

[B5923861] Reveal J. L., Utech F. H., Flora of North America Editorial Committee eds. 1993+ (2002). *
Eremocrinum
*. Flora of North America North of Mexico.

[B5924577] Reveal J. L., Flora of North America Editorial Committee eds. 1993+ (2005). *
Eriogonum
*. Flora of North America North of Mexico.

[B5535706] Riser II J. P., Cardinal-McTeague W. M., Hall J. C., Hahn W. J., Sytsma K. J., Roalson E. H. (2013). Phylogenetic relationships among the North American cleomoids (Cleomaceae): A test of Iltis's reduction series. American Journal of Botany.

[B5535694] Roalson E. H., Hall J. C., Riser II J. P., Cardinal-McTeague W. M., Cochrane T. S., Sytsma K. J. (2015). A revision of generic boundaries and nomenclature in the North American cleomoid clade (Cleomaceae). Phytotaxa.

[B5661325] Rollins R. C. (1939). The Cruciferous genus *Stanleya.*. Lloydia.

[B5532246] Rollins Reed. C. (1980). Another cruciferous weed establishes itself in North America. Contributions from the Gray Herbarium of Harvard University.

[B5700450] Sawyer Benjamin D., Hancock P. A., Deaton John, Suedfeld Peter (2012). Finding the team for Mars: a psychological and human factors analysis of a Mars Desert Research Station crew. Work.

[B5924770] Schenk J. L., Hufford L., Flora of North America Editorial Committee eds. 1993+ (2016). Mentzelia
Sect.
Bartonia. Flora of North America North of Mexico.

[B5704669] Shryock D. F., Esque T. C., Hughes L. (2014). Population viability of *Pediocactus
bradyi* (Cactaceae) in a changing climate. American Journal of Botany.

[B5353404] Shultz L. M., Neely E. E., Tuhy J. S. (1987). Flora of the Orange Cliffs of Utah. The Great Basin Naturalist.

[B5535082] Simpson B. B., Tate J. A., Weeks A. (2004). The biogeography of *Hoffmannseggia* (Leguminosae, Caesalpinioideae, Caesalpinieae): a tale of many travels. Journal of Biogeography.

[B5535092] Simpson B. B., Ulibarri E. A. (2006). A synopsis of the genus *Hoffmannseggia* (Leguminosae). Lundellia.

[B5211604] Sokoloff P. C., Freebury C. E., Hamilton P. B., Saarela J. M. (2016). The "Martian" flora: new collections of vascular plants, lichens, fungi, algae, and cyanobacteria from the Mars Desert Research Station, Utah. Biodiversity Data Journal.

[B5570462] Soreng R. J., Armstrong Wayne P., Tiehm A., Todsen Thomas K. (1984). Noteworthy collections. Madroño.

[B5353443] Spencer Tomb A (1972). Re-establishment of the genus *Prenanthella* Rydb. (Compositae: Cichorieae). Brittonia.

[B5700401] Stoker Carol R., Clarke Jonathan, Direito Susana O. L., Blake David, Martin Kevin R., Zavaleta Jhony, Foing Bernard (2011). Mineralogical, chemical, organic and microbial properties of subsurface soil cores from Mars Desert Research Station (Utah, USA): phyllosilicate and sulfate analogues to Mars mission landing sites. International Journal of Astrobiology.

[B5357509] Strother J. L. (1974). Taxonomy of *Tetradymia* (Compositae: Senecioneae). Brittonia.

[B5924864] Strother J. L., Flora of North America Editorial Committee eds. 1993+ (2006). *
Tetradymia
*. Flora of North America North of Mexico.

[B5488359] Sun Feng-Jie, Levin G. A., Downie S. R. (2005). A multivariate analysis of *Cymopterus
glomeratus*, formerly known as *C.
acaulis* (Apiaceae). Rhodora.

[B5670862] Group The Angiosperm Phylogeny (2016). An update of the Angiosperm Phylogeny Group classification for the orders and families of flowering plants: APG IV. Botanical Journal of the Linnean Society.

[B5700414] Thiel C. S., Ehrenfreund P., Foing B., Pletser V., Ullrich O. (2011). PCR-based analysis of microbial communities during the EuroGeoMars campaign at Mars Desert Research Station, Utah. International Journal of Astrobiology.

[B5661345] Turner B. L. (2004). A new variety of *Stanleya
pinnata* (Brassicaceae) from the Big Bend Region of Trans-Pecos, Texas. Lundellia.

[B5704996] USDA NRCS The PLANTS Database. http://plants.usda.gov.

[B5663004] Resources Utah Division of Wildlife (1998). Inventory of sensitive species and ecosystems in Utah.

[B5924757] Vanderpool S. S., Flora of North America Editorial Committee eds. 1993+ (2010). *
Cleomella
*. Flora of North America North of Mexico.

[B5566012] Walden G. K., Garrison L. M., Spicer G. S., Cipriano F. W., Patterson R. (2014). Phylogenies and chromosome evolution of *Phacelia* (Boraginaceae: Hydrophylloideae) inferred from nuclear ribosomal and chloroplast sequence data. Madroño.

[B5566002] Walden G. K., Garrison L. M., Patterson R. (2016). Nomenclatural adjustments in Phacelia
sect.
Glandulosae (Hydrophyllaceae, Boraginales). Western North American Naturalist.

[B5224325] Welsh S. L., Atwood N. D., Goodrich S, Higgins L. C. (1993). A Utah flora.

[B5353463] Welsh S. L. (2003). North American apecies of *Atriplex* Linnaeus (Chenopodiaceae): a taxonomic revision.

[B5353414] Welsh S. L. (2006). North American species of *Astragalus* Linneaus (Leguminosae): a taxonomic revision.

